# Effect of neuromuscular electrical stimulation in critically ill adults with mechanical ventilation: a systematic review and network meta-analysis

**DOI:** 10.1186/s12890-024-02854-9

**Published:** 2024-01-25

**Authors:** Cuiping Xu, Feng Yang, Qimin Wang, Wei Gao

**Affiliations:** Department of Respiratory and Critical Care Medicine, China Rehabilitation Research Center, Rehabilitation School of Capital Medical University, Beijing, China

**Keywords:** Neuromuscular electrical stimulation, Physical rehabilitation, Mechanical ventilation, Network meta-analysis

## Abstract

**Background:**

Neuromuscular electrical stimulation (NMES) is widely used as a rehabilitation methods to restore muscle mass and function in prolonged immobilization individuals. However, its effect in mechanically ventilated patients to improve clinical outcomes remains unclear.

**Methods:**

A comprehensive search was conducted using PubMed, Embase, Web of Science, PEDro, and the Cochrane Library from their inception until December 24th, 2023. The search targeted randomized controlled trials (RCTs) comparing NMES with physical therapy (PT) or usual ICU care (CG), for improving clinical outcomes in mechanically ventilated patients. We performed a network meta-analysis utilizing Stata version 14.0 and R 4.3.1.

**Results:**

We included 23 RCTs comprising 1312 mechanically ventilated adults. The treatments analyzed were NMES, PT, NMES combined with PT (NMES+PT), and CG. Network meta-analyses revealed that NMES or NMES+PT significantly improved extubation success rate compared to CG, with ORs of 1.85 (95% CI: 1.11, 3.08) and 5.89 (95% CI: 1.77, 19.65), respectively. Additionally, NMES exhibited a slight decrease in extubation success rate compared with NMES+PT, with OR of 0.31 (95% CI: 0.11, 0.93). Nevertheless, neither NMES nor NMES+PT showed any significant improvement in ICU length of stay (LOS), ventilation duration, or mortality when compared with PT or CG. NMES+PT emerged as the most effective strategy for all considered clinical outcomes according to the ranking probabilities. The evidence quality ranged from “low” to “very low” in this network meta-analysis.

**Conclusions:**

NMES appears to be a straightforward and safe modality for critically ill, mechanically ventilated patients. When combined with PT, it significantly improved the extubation success rate against standard ICU care and NMES alone, and showed a better ranking over PT or NMES alone for clinical outcomes. Therefore, NMES combined with PT may be a superior rehabilitation strategy for this patient group.

**Supplementary Information:**

The online version contains supplementary material available at 10.1186/s12890-024-02854-9.

## Background

Critically ill patients often experience prolonged periods of bed rest and inactivity resulting from their stay in the intensive care unit (ICU), and this is particularly true for those requiring mechanical ventilation (MV) [1]. Such patients are at a heightened risk of rapid muscle mass deterioration, with approximately 37% exhibiting signs of muscle atrophy as early as the fourth day of their ICU admittance [2]. This loss of skeletal muscle is correlated with diminished physical capabilities, extended duration of mechanical ventilation, prolonged ICU stays, and an increase in mortality [3]. Therefore, it is essential to implement immediate strategies to mitigate muscle degradation as promptly as possible for these individuals.

Early physical rehabilitation, including early mobility and exercise during the initial days of ICU admission for ventilated patients, stands as a crucial method to influence or even prevent skeletal muscle debilitation and atrophy [4–7]. Studies have shown that early physiotherapy protocol can enhance muscle quality and functionality in critically ill patients, as well as decrease length of ICU stay and mechanical ventilation duration [8, 9]. Nonetheless, early and intensive mobilization in ventilated patients presents challenges due to their severe conditions, high levels of ventilatory support, or impaired consciousness [10, 11]. In the early stages, feasible exercises for these patients typically involve low-intensity or passive activities, such as passive cycling at the bedside [12]. Another obstacle to early active rehabilitation is the potential insufficient duration of available physiotherapy time, often due to the lack of physiotherapists. Consequently, there is a need for alternative or supplemental rehabilitation methodologies that do not rely on patient cooperation or that utilize automated devices.

Neuromuscular electrical stimulation (NMES) is a technique that utilizes an automated device to apply surface electrodes on the skin, which activates intramuscular nerve branches and induces visible muscle contractions [13]. It has been demonstrated to be effective in mitigating muscle loss and enhancing muscle strength in patients requiring MV [14, 15]. Nakanishi N. et al. investigated the use of NMES in mechanically ventilated patients and found that it could prevent both upper and lower limb muscle atrophy and reduce the duration of hospitalization [16]. Furthermore, NMES is well-tolerated and does not necessitate patient cooperation.

To date, several systematic reviews have been conducted to assess the effects of NMES on critically ill or ventilated patients [17–23], however, these studies report conflicting results. Certain meta-analyses have shown that NMES application can not only effectively shorten the duration of ventilation but also ameliorate the functional status of mechanically ventilated patientss [17, 18, 23], while others have not found such effects, noting that NMES combined with standard care did not yield significant benefits in terms of muscle strength, ventilation duration, ICU mortality, or ICU length of stay (LOS) when compared to standard care alone [21].

These discrepancies could be attributed to varying inclusion criteria and publication dates of the reviews. Another possible contribution to the inconsistency is the heterogeneity of the interventions of the interventions among the included trails. In some trials within the experimental group, NMES was used alone, while in others it was combined with physical therapy. Conversely, some trials employed physical therapy as a control method, while others applied standard ICU care without exercise. Such variability makes it difficult to determine the true effect of NMES.

Therefore, we conducted this systematic review and network meta-analysis, focusing on different rehabilitation strategies, to determine whether NMES application can improve clinical outcomes such as ICU LOS, ventilation duration, extubation success rate or mortality in mechanically ventilated patients.

## Methods

This systematic review was conducted according to the Preferred Reporting Items for Systematic Reviews and Meta-Analyses for Network Meta-Analyses (PRISMA-NMA) [24] (Supplementary Table 1 for the detailed PRISMA-NMA Checklist of this study).

### Eligibility criteria

We searched for RCTs in critically ill adult patients with MV, which investigated NMES as a rehabilitation intervention, comparing with other interventions such as physical therapy (PT), NMES combined with PT (NMES+PT) or usual ICU care (CG).

Inclusion criteria for the studies were as follows: (1)Population: Adult patients(≥ 18 years of age) admitted to the ICU who required MV via either an endotracheal tube or tracheotomy; (2) Intervention and comparisons: The primary intervention assessed was NMES, either independently or in combination with PT, with comparisons including PT alone or usual ICU care; (3) Study Design: Only RCTs were included; (4) Outcomes: Studies needed to report on at least one of the following clinical outcomes: ICU LOS, ventilation duration, extubation success rate, and mortality within the ICU or hospital.

### Search strategy

Two reviewers (CPX and QMW) independently conducted comprehensive searches of PubMed, Embase, Web of Science, PEDro, and the Cochrane Library databases from their inception to December 24th, 2023, without language or publication type restrictions. Additionally, we examined the reference lists of all pertinent articles and the citations within previously published meta-analyses to identify further potential studies (Supplementary Table 2 for the details of the search strategy).

### Studies selection

According to the inclusion criteria, two reviewers (CPX and QMW) independently screened the titles and abstracts of the retrieved studies, and the full text was assessed as necessary, to identify the eligible studies. Any disagreements were resolved by discussing with a third researcher (WG) to reach a consensus.

### Data extraction

A standardized data extraction form was utilized to systematically collect data from every study included in the analysis. We extracted details of study information such as the first author’s name, year of publication, the country or region of the study, setting, sample size, duration of the study, and intervention methods. Furthermore, we extracted participant demographics and baseline clinical measurements, including age, the baseline Acute Physiology and Chronic Health Evaluation II (APACHE II) score, and baseline body mass index (BMI). Clinical outcomes were also recorded, encompassing ventilation duration, extubation success rate, ICU LOS, ICU or hospital mortality. The two investigators undertook the data extraction process independently; conflicts were again resolved through consultation with WG. Additionally, supplemental files were reviewed and the authors of the articles were contacted for further details as needed.

### Quality assessment

Two reviewers (CPX and QMW) assessed the studies’ risk of bias according to Cochrane risk of bias tool (ROB tool) independently. The tool included seven different items: (1) random sequence generation, (2) allocation concealment, (3) blinding of participants and personnel, (4) blinding of outcome assessment, (5) incomplete outcome data, (6) selective reporting, and (7) other sources of bias. Based on the methods of the trial, each item of the ROB was judged as “high risk”, “low risk”, or “unclear risk”. Additionally, we assessed the quality of evidence contributing to network estimates of the four outcomes with the Grading of Recommendations Assessment, Development and Evaluation (GRADE) framework [25, 26]. Two reviewers (CPX and QMW) made judgments independently, and disagreements were resolved through discussion with a third reviewer (GW) to reach an agreement.

### Statistical synthesis and analysis

The statistical analyses were performed using Stata version 14.0 and R 4.3.1. A random-effects model was applied to both pairwise meta-analyses and network meta-analyses. Odds ratios (ORs) with 95% confidence intervals (CIs) were utilized to estimate the effects of dichotomous variables, whereas mean differences (MDs) with 95% CIs were used for continuous variables. The I [2] statistic was calculated to quantify heterogeneity, representing the proportion of total variation attributable to between-study differences. Ranking probabilities for each intervention’s outcomes were calculated and expressed as the surface under the cumulative ranking curve (SUCRA) and visualized using cumulative ranking plots. The SUCRA provides a numerical representation of each intervention’s overall and mean rank, ranging from 0 to 1, with higher values indicating superior rankings.

To assess global inconsistency across the entire analytical network, we employed a design-by-treatment interaction approach. Local inconsistency was appraised using both loop-specific approaches and the node-splitting method. Global heterogeneity was evaluated using the I^2^ statistic, local heterogeneity was assessed by predictive interval plots, where discrepancies between the confidence intervals of relative treatment effects and their predictive intervals indicated uncertainty due to heterogeneity. Furthermore, a comparison-adjusted funnel plot was utilized to investigate potential publication bias in the included studies. A contribution plot highlighted the influence of each direct comparison on the estimation of each network meta-analytic summary effect. We also conducted a sensitivity analysis by excluding two trials that investigated NMES in patients with prolonged mechanical ventilation to gauge the robustness of the results for the four clinical outcomes examined.

## Results

### Literature identification and selection

**Fig. 1:**
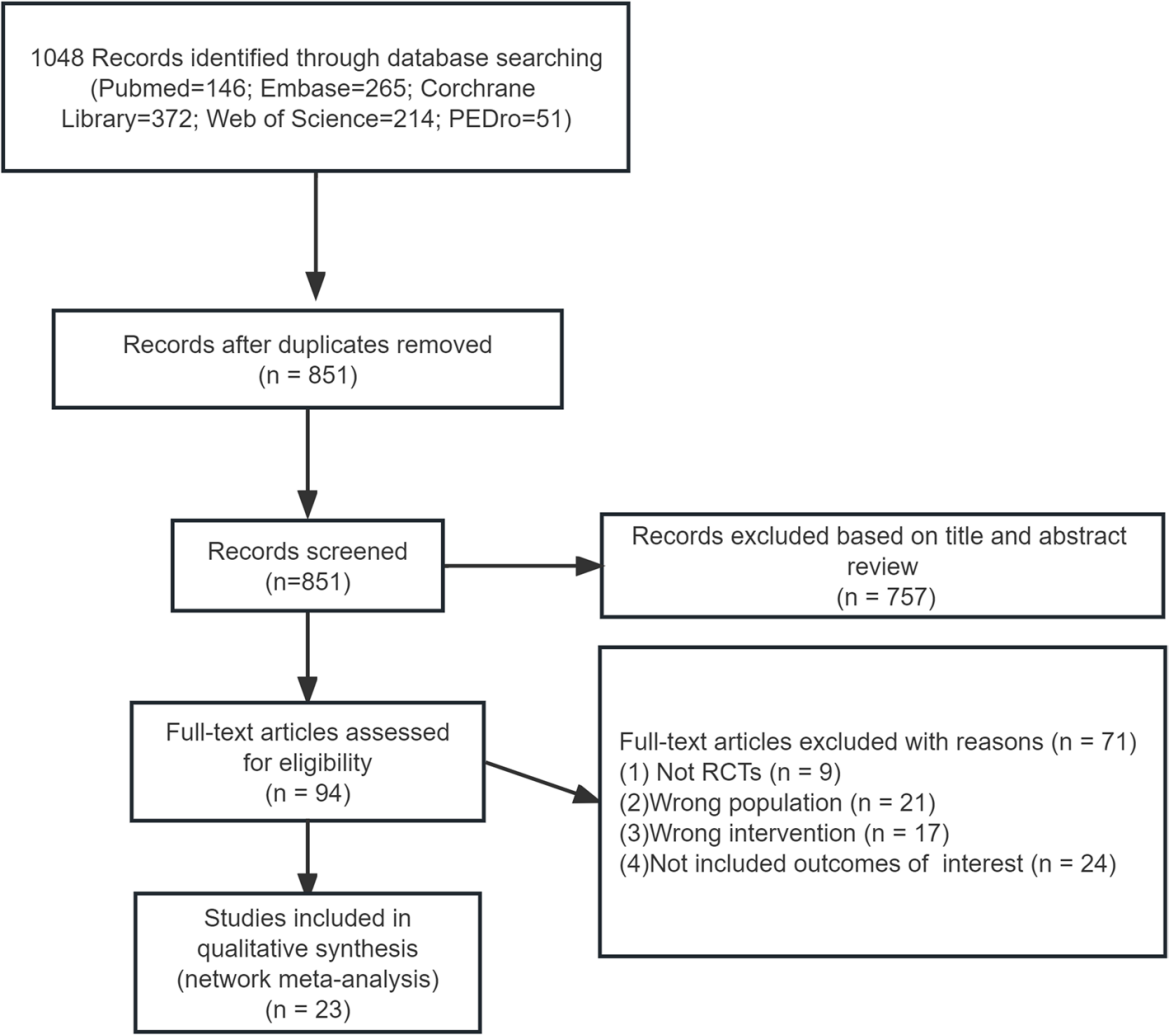
Flow chart of the trial selection process

From the initial literature search, we identified a total of 1048 citations (PubMed, *n* = 146; Embase, *n* = 265; Cochrane Library, *n* = 372; Web of Science, *n* = 214; PEDro, *n* = 51). Following duplicate removal, 851 citations were screened by titles and abstracts. Subsequently, 757 articles were excluded based on the eligibility criteria. We evaluated the full texts of the remaining 94 articles, with 23 RCTs ultimately meeting the inclusion criteria (A flow chart of the trial selection process is presented in Fig. 1).

### Study characteristics

**Fig. 2:**
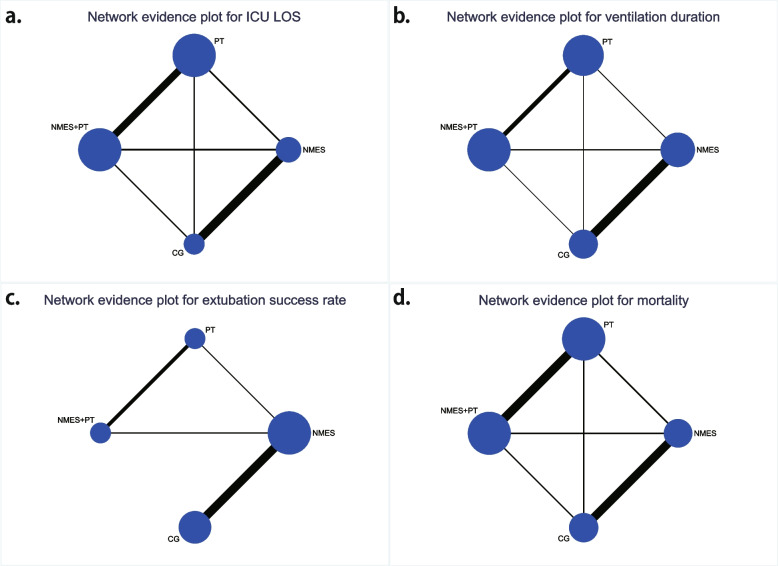
Network evidence plots of eligible comparisons for network meta-analysis. a. ICU LOS; b. ventilation duration; c. extubation success rate; d. mortality

The systematic review included 23 RCTs [2, 16, 27–47], of which 21 were published in English-language journals and two in Chinese-language journals. Publication years ranged from 2012 to 2023, and intervention durations varied from 7 days to 6 months. Twenty trials were two-arm studies, while three were multiple-arm studies. A total of 1163, 1145, 552, and 752 patients contributed to the respective clinical outcomes of ICU LOS, ventilation duration, extubation success rate, and mortality. Analyses were conducted on four interventions: NMES, PT, NMES+PT, and CG. Among the included trials, eight compared NMES with CG, twelve compared NMES+PT with PT, one compared NMES with both PT and NMES+PT, and two compared all four treatments (The network evidence plots for this study are shown in Fig. 2). Overall, 91.3% (21/23) investigated NMES in the early stages of mechanical ventilation, with males comprising 63.9% (838/1312) of participants. The mean age of participants was 53.89 ± 18.85 years (mean ± SD), the baseline mean BMI was 25.16 ± 6.07 kg/m^2^ (mean ± SD), and the baseline mean APACHE II score was 19.58 ± 7.56 (mean ± SD). Fifteen trials applied NMES to the quadriceps muscle, either alone or in combination with other muscle groups, five applied NMES to the abdominal muscle, either alone or in combination with the diaphragm, and three trails applied NMES to the diaphragm alone. Five trials reported the time from ICU admission to the first NMES intervention session, ranging from 2 hours to 4.6 ± 1.8 days (mean ± SD), with a mean duration of 2.5 ± 1.8 days (mean ± SD). Significant heterogeneity existed within the studies in terms of stimulation parameters. For example, for quadriceps muscles, the stimulation frequency ranged from 30 Hz to 100 Hz, with most trials (8/13) employing 50 Hz; and the pulse width applied varied from 200 μs to 500 μs, with the majority (7/11) using 400 μs. For the diaphragm or abdominal muscles, most trials utilized a frequency of 30 Hz, with only one using 50 Hz, and the pulse duration applied ranging from 300 μs to 400 μs. All studies utilized a stimulation intensity capable of eliciting a visible muscle contraction. Among the included trials, only a few reported a low number of adverse events related to NMES, including discomfort, prickling sensations, and brief, spontaneous reversible episodes of hypertension or tachycardia. No serious NMES-related adverse events were reported (The detailed characteristics of the included trials are provided in Table 1).

**Table 1:** The characteristics of included studies

Study	Patients	Number of participants	Age (years), mean ± SD	Baseline BMI (kg/m^2^), mean ± SD	Baseline APACHE II, mean ± SD	Application of NMES	Measurement timepoint	outcomes
McCaughey EJ, et al. 2019	critically ill mechanically ventilated participants	NMES = 10 CG = 10	58.75 ± 15.33	NR	NR	NMES for 30 min, twice daily, 5 days per week, until ICU discharge. NMES was on the dominis and internal and external oblique muscles, with stimulation frequency of 30 Hz, pulse width of 350 us, and at an intensity was able to cause a strong visible muscle contraction (median 60 mA [range 50–65 mA])	ventilation duration, ICU LOS, ICU mortality, extubation success rate.	6-month
Jonkman AH, et al. 2020	mixed ICU ventilated patients within 72 hours after intubation	NMES = 10 CG = 10	68.95 ± 8.51	26.35 ± 4.40	NR	NMES was on the abdominal wall for 30 min twice daily, 5 days per week, until patients were weaned from mechanical ventilation, but no longer than 6 weeks, with stimulation frequency of 30 Hz, pulse width of 352 us, and at an intensity was able to cause strong muscle contraction, with a maximum intensity initially set at 60–100 mA.	ventilation duration, ICU LOS, and ICU mortality	until weaned from MV, but shorter than 6 weeks
Abu-Khaber HA, et al. 2013	ICU patients with MV for more than 24 hours	NMES = 40 CG = 40	58.32 ± 6.11	NR	25.3 ± 6.11	NMES was simultaneously on the quadriceps muscles of both lower extremities for 1 h once daily, starting from the second day after admission until ICU discharge, with stimulation frequency of 50 Hz, pulse width of 200 μs and at intensity able to cause visible contractions (mostly 100–150 MA).	ventilation duration, 28 days mortality, extubation success rate	28 days or until the time of weaning from MV
Study	Patients	Number of participants	Age (years), mean ± SD	Baseline BMI (kg/m^2^), mean ± SD	Baseline APACHE II, mean ± SD	Application of NMES	Measurement timepoint	outcomes
Koutsioumpa et al. 2018	Adult patients use of mechanical ventilation for 96 hours or more.	NMES+PT = 38 PT = 42	65.05 ± 12.73	27.00 ± 6.51	19.1 ± 7.97	NMES was simultaneously on the quadriceps muscle of each lower extremity for 1 h once daily from the 4th to the 14th ICU Day, with impulses of 50 Hz per 500 ms phase duration, at intensities able to cause visible muscle contractions.	ventilation duration, 28-day mortality.	10 days
Hsin YF, et al. 2022	Participants on ventilation for ≥21 days	NMES = 29 CG = 30	75.23 ± 10.50	22.76 ± 4.62	18.39 ± 3.97	Transcutaneous electrical diaphragmatic stimulation intervention for 30 min/day, 5 days/week until the end of the weaning trial, with stimulation frequency of 30 Hz, pulse width of 400 us, at an intensity was gradually increased until visible muscle contraction was observed.	ventilation duration, mortality, extubation success	the end of the weaning trial
Chen YH, et al. 2019	Participants on ventilation for ≥21 days	NMES = 16 CG = 17	75.69 ± 16.07	23.00 ± 4.72	20.54 ± 6.75	NMES was on motor points of the vastus lateralis and rectus femoris of both legs, for 30-min twice per day, 5 days per week for 2 weeks, with simulation frequency of 50 Hz, pulse width of 400 us, and the intensity was gradually increased until a visible muscle contraction was observed.	ventilation duration, mortality, extubation success	2 weeks
Study	Patients	Number of participants	Age (years), mean ± SD	Baseline BMI (kg/m^2^), mean ± SD	Baseline APACHE II, mean ± SD	Application of NMES	Measurement timepoint	outcomes
Leite MA, et al.2018	Patients on MV for 24 hours or more.	NMES+PT = 41 PT = 26	44.41 ± 18.72	23.34 ± 8.52	18.57 ± 3.23	Subjects received NMES were randomized into two groups: diaphragm group (DG) or quadriceps group (QG). NMES was performed on the right and left sides of the xiphoid process within the seventh and eighth anterior intercostal space, with a frequency of 30 Hz for DG, and on quadriceps with frequency of 50 Hz for QG. Each session was performed for 45 min daily at intensities that produced visible contractions.	ventilation duration, ICU length of stay	NR
Shen SY, et al. 2017	Patients required MV longer than 72 hours	NMES+PT = 18 PT = 7	77.64 ± 6.73	NR	23.92 ± 6.08	NMES was on both quadriceps and biceps, for 32 minutes per day, 5 days per week. The stimulator output current was 0–75 mA in biphasic waves with carrier frequency of 1500 Hz. The lowest stimulation current was given to induce visible muscle contraction.	ventilation duration, hospital mortality, extubation success	NR
Dall’ Acqua AM, et al. 2017	Patients hospitalized for no longer than 15 days and had received MV for 24–48 hours	NMES+PT = 11 PT = 14	58.8 ± 14.09	24.44 ± 4.52	27.68 ± 6.27	NMES was performed on the chest muscles (pectoralis major muscle fibers) and rectus abdominis muscles bilaterally, for 30 min once a day, with frequency of 50 Hz, pulse duration of 300 us, and the intensity was increased until muscle contraction was visible or could be identified through palpation.	ventilation duration, ICU LOS, mortality	day 7 of MV or 24 h after extubation
Study	Patients	Number of participants	Age (years), mean ± SD	Baseline BMI (kg/m^2^), mean ± SD	Baseline APACHE II, mean ± SD	Application of NMES	Measurement timepoint	outcomes
Kho ME, et al.2015	Patients received MV within the first week of ICU stay	NMES+PT = 16 PT = 18	55.06 ± 16.86	27 ± 7.05	25 ± 6.90	NMES was on quadriceps, tibialis anterior, and gastrocnemius bilaterally, for 60 minutes daily, with frequency of 50 Hz, pulse duration of 250 us for the tibialis anterior and gastrocnemius, 400us for the quadriceps, at intensities able to cause visible muscle contractions.	ventilation duration, ICU LOS, hospital mortality	at hospital discharge
Dos Santos FV, et al.2020	Adults received MV for less than 72 hours, without neuromuscular disease.	NMES = 11 CG = 15 NMES+PT = 12 PT = 13	53.24 ± 12.17	NR	15.90 ± 3.54	NMES was on the rectus femoris, vastus lateralis, and vastus medialis muscles bilaterally, for 55 minutes twice a day, 7 days per week, with frequency of 45 Hz, pulse duration of400 μs, at intensities able to cause visible muscle contractions.	ventilation duration, ICU LOS, mortality	Until discharged from ICU, for a maximum duration of 6 weeks
Silva PE, et al.2019	Critically ill traumatic brain injury adults received MV for up to 24 hours	NMES+PT = 30 PT = 30	31.5 ± 11.17	NR	11 ± 3.7	NMES was on the lower limb muscles for a 30-minute session daily for 14 consecutive days, with frequency of 100 Hz, pulse duration of 400 us, at intensities to evoke maximum contractions in each muscle group.	ventilation duration, ICU LO, ICU mortality	14 days
Study	Patients	Number of participants	Age (years), mean ± SD	Baseline BMI (kg/m^2^), mean ± SD	Baseline APACHE II, mean ± SD	Application of NMES	Measurement timepoint	outcomes
Mahran GSK, et al. 2023	Adults required MV on the first day of admission	NMES+PT = 60 PT = 58	31.49 ± 9.49	NR	13.82 ± 6.09	NMES was on abdominal muscles for 40 minutes(20 minutes on diaphragmatic muscles and 20 minutes to the abdominal muscles) daily until the seventh day of admission, with frequency of 20 Hz, at intensities able to cause visible muscle contractions.	ventilation duration, ICU LOS, mortality	the seventh day of admission
Chen S, et al.2019	COPD patients admitted to ICU and received MV	NMES+PT = 27 PT = 29	40.34 ± 23.63	22.37 ± 1.53	19.41 ± 4.30	NMES was on the extremities for 30 minutes twice a day after 24 hours of admission until ICU discharge, with frequency of 30 ~ 40 Hz.	ventilation duration, ICU LOS	until ICU discharge
Peng, Lu, et al.2022	ICU patients received MV for more than 24 hours	NMES = 30 PT = 30 30 NMES+PT = 30	58.25 ± 14.63	19.77 ± 6.22	NR	Patients received NMES of 30 min/day, 5 days/week for 2 weeks on gastrocnemius muscles and abdominis muscles.	extubation success rate, ICU LOS, ventilation duration	2 weeks
Verceles AC, et al.2023	Older patients (> 50 years) received MV for more than 24 hours	NMES+PT = 16 PT = 23	62 ± 9.2	29.59 ± 6.12	16.38 ± 6.71	NMES was applied to the quadriceps for 30 minutes twice daily for 10 days, with frequency of 50 Hz, pulse duration of 300 us, at intensities able to cause visible muscle contractions.	ventilation duration, ICU LOS	14 days
**Study**	**Patients**	**Number of participants**	**Age (years), mean ± SD**	**Baseline BMI (kg/m**^**2**^**), mean ± SD**	**Baseline APACHE II, mean ± SD**	**Application of NMES**	**Measurement timepoint**	**outcomes**
Medrinal C,et al.2023	Adults ventilated for at least 24 hours	NMES = 30 CG = 31	62 ± 12.6	27 ± 5.47	NR	NMES was applied on diaphragm for 20 minutes daily, 5 times per week, with frequency of 50 Hz, pulse duration of 300 us, at intensities able to cause visible muscle contractions.	ventilation duration, ICU LOS, extubation success rate	until the first extubation attempt
Liu Y, et al.2023	Patients on MV for respiratory failure	NMES+PT = 40 PT = 40	58.62 ± 15.69	24.51 ± 4.69	20.17 ± 6.39	NMES was applied on the pectoralis major fibers, rectus abdominis and bilateral quadriceps muscles, for 30 minutes per day until discharged from ICU, with frequency of 50 Hz, pulse duration of 300 us, at intensities gradually increase until significant muscle contractions occur.	ventilation duration, ICU LOS, extubation success rate, mortality	at discharge from ICU
Campos DR, et al.2022	Adults less than 48 hours of ICU admission and an expected stay on MV for longer than 48 hours	NMES+PT = 34 PT = 40	44.77 ± 6.61	26.46 ± 5.50	NR	NMES was applied on the quadriceps and the tibialis anterior, once a day for 60 minutes, 5 days a week, from ICU admission until ICU discharge, with frequency of 80 Hz, pulse duration of 400 us, at intensities able to cause visible muscle contractions, and the maximum intensity was 120 mA.	ventilation duration, ICU LOS	until ICU discharge
Othman SY, et al.2023	Adults on MV and was supposed to be on MV for longer than 48 hours	NMES = 30 PT = 30 NMES+PT = 30 CG = 30	41.77 ± 0.87	30.08 ± 2.44	29.05 ± 3 .95	NMES was applied on the quadriceps for 60 min daily, from day 2 of ICU admission to day 7, with frequency of 50 Hz, pulse duration of 400 us, at intensities able to cause visible muscle contractions.	ventilation duration	7 days
**Study**	**Patients**	**Number of participants**	**Age (years), mean ± SD**	**Baseline BMI (kg/m**^**2**^**), mean ± SD**	**Baseline APACHE II, mean ± SD**	**Application of NMES**	**Measurement timepoint**	**outcomes**
Olimpio JH, et al.2023	Patients aged ≥60 years and were on MV for at least 24 hours	NMES = 21 CG = 25	66.63 ± 5.22	NR	NR	NMES was applied on the diaphragm for 30 minutes twice a day immediately after the 24-h period on IMV until extubation in spontaneous ventilatory mode, with frequency of 30 Hz, pulse duration of 400 us, at intensities able to cause visible muscle contractions.	ventilation duration, extubation success rate	until extubation in spontaneous ventilatory mode
Nakanishi N, et al.2020	Adult patients who were expected to be on MV for > 48 hours	NMES+PT = 17 PT = 19	69.31 ± 4.62	23.53 ± 3.97	23.42 ± 8.18	NMES was applied on the bilateral upper and lower limbs for 30 min daily from day 1 to day 5, with frequency of 20 Hz, at intensities able to cause visible muscle contractions. The EMS intensities used were 30 mAp (23–37 mAp) for the biceps brachii and 41 mAp (33–50 mAp) for the rectus femoris.	ventilation duration, ICU LOS	discharge from the ICU
Vieira L, et al.2023	Patients on MV secondary to traumatic brain injury	NMES = 20 CG = 20	35.6 ± 12.28	NR	16.4 ± 4.50	NMES was applied on the quadriceps muscles for 55 minutes daily for five consecutive days, with frequency of 50 Hz, pulse duration of 400 us, at intensities able to evoke maximum muscle contraction.	ventilation duration, ICU LOS, extubation success rate, mortality	7 days

*ICU* intensive care unit: *LOS* length of stay: *NMES* neuromuscular electrical stimulation: *PT* physical therapy: *NMES + PT* NMES combined with PT: *CG* usual *ICU* care: *SD* standard deviation: *APACHE II* Acute Physiology and Chronic Health Evaluation II: *BMI* body mass index: *MV* mechanical ventilation: *COPD* chronic obstructive pulmonary disease: *N* number: *NR* not reported

### Risk of bias assessment

**Fig. 3:**
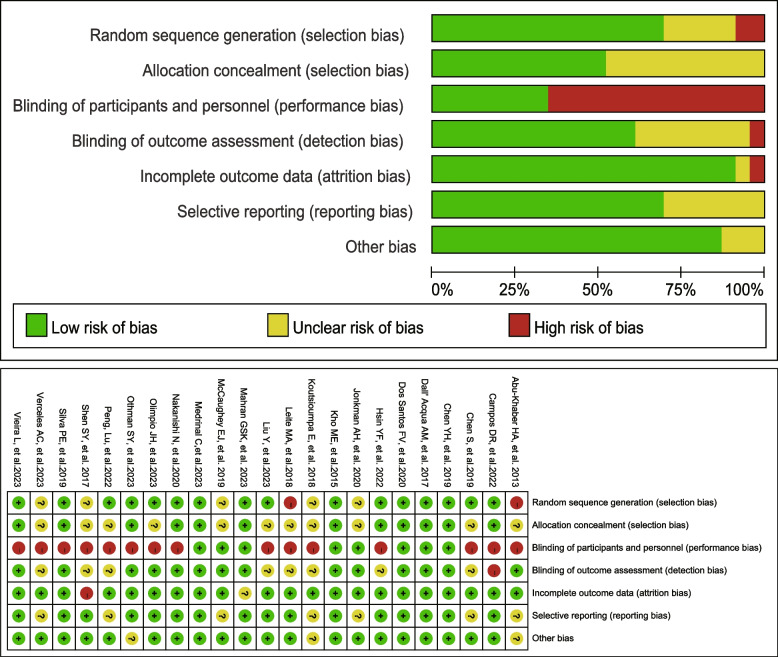
Results of risk assessment of bias using Cochrane risk of bias tool

According to the ROB tool, five trials did not adequately describe their randomization methods, and two trials exhibited a high risk of bias within this domain. Furthermore, eleven trials failed to clearly report allocation concealment procedures, and fifteen trails did not achieve blinding of participants. Conversely, fourteen trials demonstrated a low risk of bias concerning blinding of outcome assessment, while eight trails displayed an unclear risk in this area, and the remain one had a high risk of bias in this domain.. 21 trials reported complete outcome data, one trial suffered from incomplete outcome data, and another lacked clarity regarding outcome data completeness. Selective reporting was absent in sixteen studies, and the majority of studies (20 out of 23) presented a low risk of bias in other sources of bias, with only three failing to disclose their funding sources. Ultimately, 15 studies were regarded to have a high risk of bias, whereas only 5 were assessed as having a low risk (The risk of bias assessment is provided in Fig. 3 and Supplementary Table 3).

### Results of pairwise meta-analysis and network meta-analysis

**Fig. 4:**
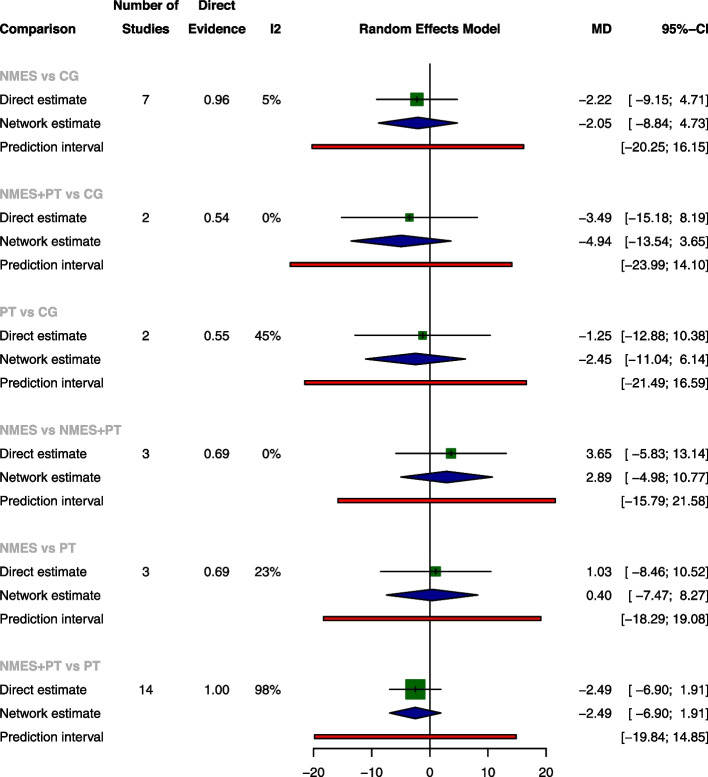
Forest plot of pairwise meta-analysis, network meta-analysis and predictive interval for ICU LOS. Both the pairwise meta-analysis and the network meta-analysis revealed no significant differences among the four treatments for ICU LOS. Predictive interval plots suggested no significant heterogeneity in the network meta-analysis among the comparisons for ICU LOS

**Fig. 5:**
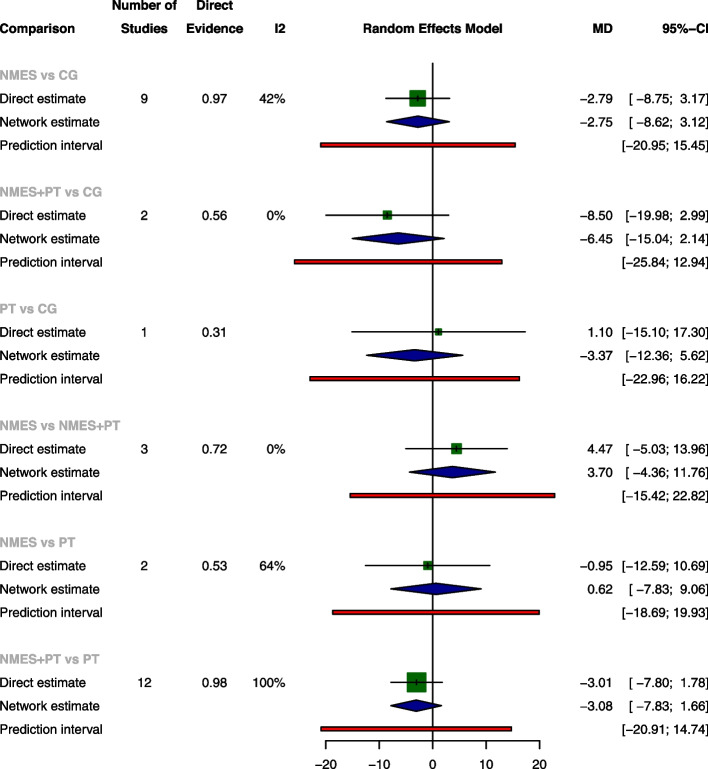
Forest plot of pairwise meta-analysis, network meta-analysis and predictive interval for ventilation duration. Both the pairwise meta-analysis and the network meta-analysis revealed no significant differences among the four treatments for ventilation duration. Predictive interval plots suggested no significant heterogeneity in the network meta-analysis among the comparisons for ventilation duration

**Fig. 6:**
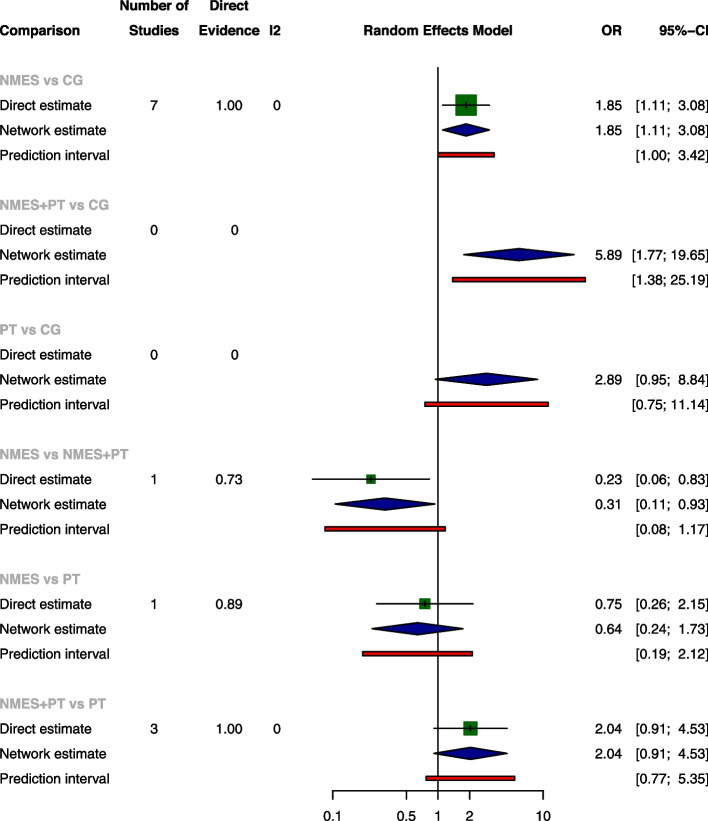
Forest plot of pairwise meta-analysis, network meta-analysis and predictive interval for extubation success rate. The pairwise meta-analysis illustrated significant improvement in extubation success rate with NMES compared to CG. However, the combination of NMES and Physical Therapy (NMES+PT) displayed a slightly higher success rate than NMES alone. The network meta-analysis further confirmed the superiority of both NMES and NMES+PT over CG in terms of the extubation success rate. The predictive interval plots revealed significant heterogeneity in the network meta-analysis when comparing NMES with CG and NMES+PT with CG regarding the extubation success rate

**Fig. 7:**
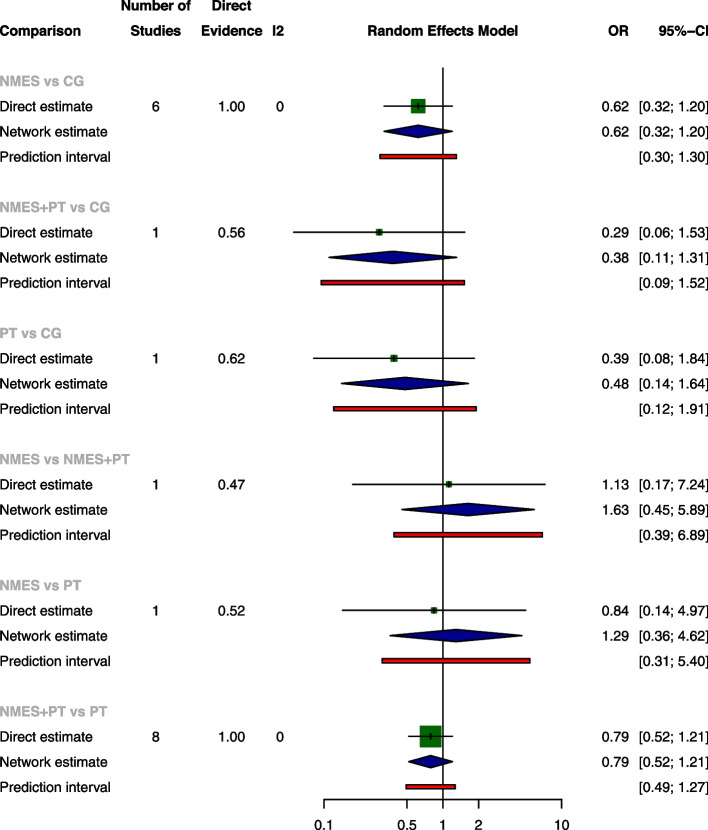
Forest plot of pairwise meta-analysis, network meta-analysis and predictive interval for mortality. Both the pairwise meta-analysis and the network meta-analysis revealed no significant differences among the four treatments for mortality. Predictive interval plots suggested no significant heterogeneity in the network meta-analysis among the comparisons for mortality

The pairwise meta-analysis revealed no significant differences among the four treatments concerning ICU LOS, ventilation duration, and mortality rates. NMES was associated with a significant increase of extubation success rate when compared with CG, with an OR of 1.85 (95% CI: 1.11, 3.08), while NMES was slightly less effective than NMES+PT, as indicated by an OR of 0.23 (95% CI: 0.06, 0.83). The network meta-analysis further established NMES and NMES+PT as superior to CG for extubation success rate, with ORs of 1.85 (95% CI: 1.11, 3.08) and 5.89 (95% CI: 1.77, 19.65), respectively. Consistent with the pairwise meta-analyses, the network meta-analysis also shown a slight decrease in extubation success rate when NMES was compared with NMES+PT, with OR of 0.31 (95% CI: 0.11, 0.93). There were no significant differences among the treatments for ICU LOS, ventilation duration, and mortality according to the network meta-analysis (The results of pairwise meta-analysis and network meta-analysis are shown in Figs. 4, 5, 6, and 7).

Examination of the network contribution plots (Supplementary Fig. 1a-1d) revealed that NMES versus NMES+PT and NMES+PT versus CG had the most substantial influence on the network for ICU LOS, with contributions of 21.7 and 20.9%, respectively. Similarly, PT versus CG and NMES+PT versus CG were predominant for ventilation duration, contributing to 23.2 and 22.2% of the network, respectively. In the context of extubation success rate, the largest impact was seen in NMES versus CG and NMES versus PT comparisons, with contributions of 30.6 and 25.5%, whereas the comparison between PT and NMES+PT, and NMES versus CG presented the most significant contributions of 28.8 and 26.6% for mortality.

### Transitivity, inconsistency and heterogeneity

No significant global inconsistency was detected by the design-by-treatment interaction model for all the four outcomes (*P* = 0.9844 for ICU LOS, *p* = 0.8107 for ventilation duration, *p* = 0.3692 for extubation success rate and *p* = 0.7168 for mortality, respectively). Local inconsistency tests corroborated these findings, indicating consistency in ICU LOS, ventilation duration, extubation success, and mortality, as evidenced by 95% CIs encompassing 0 in the inconsistency plots (Inconsistency assessment shown in Supplementary Fig. 2a-2d). The node-splitting model further supported the absence of significant differences in comparisons across all four outcomes, with *P*-values ranging from 0.205 to 0.989 (Supplementary Fig. 3a-3d). Nevertheless, predictive interval plots suggested significant heterogeneity in the network meta-analysis (NMA) when comparing NMES with CG and NMES with NMES+PT regarding the extubation success rate (Predictive interval plots shown in Figs. 4, 5, 6, and 7). The funnel plots for ICU LOS, ventilation duration and mortality were relatively symmetrical and did not suggest significant risk of publication bias among the included studies. In contrast, the publication bias was statistically significant for extubation success rate according to the funnel plot (Comparison-adjusted funnel plots shown in Supplementary Fig. 4a-4d).

### SUCRA and ranking of all interventions

**Fig. 8:**
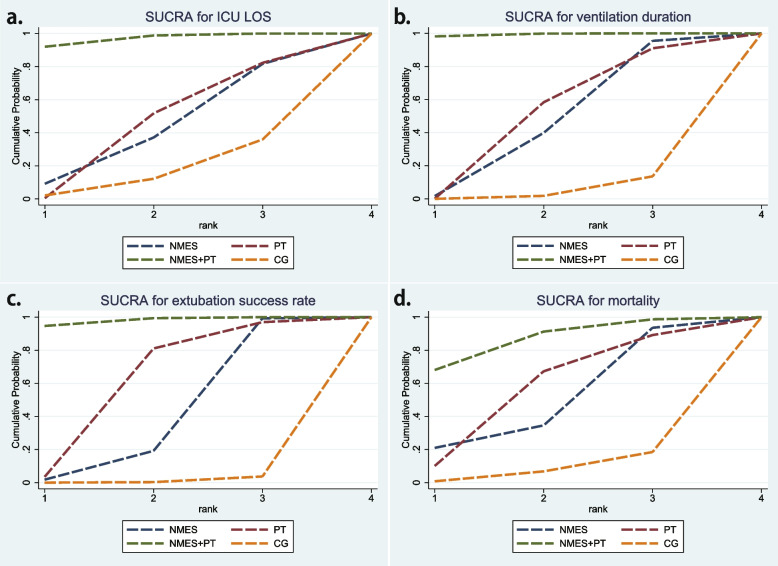
Plots of cumulative ranking probability by SUCRA for each outcome. **a** ICU LOS; **b** ventilation duration; c. extubation success rate; d. mortality

The SUCRA indicates that NMES was ranked third in effectiveness for ICU LOS, ventilation duration, extubation success rate, and mortality, with respective probabilities of 44.5, 55.6, 80.1, and 59%. PT was ranked second for each of these outcomes, with probabilities of 51.3, 58.3, 77.6, and 57.2%. NMES+PT emerged as the most effective intervention across all four outcomes, exhibiting probabilities of 92, 98.2, 94.7, and 68.1%. In contrast, CG ranked lowest for all outcomes with corresponding probabilities of 64, 86.4, 96.3, and 81.5% (Plots of cumulative ranking probability by SUCRA are depicted in Fig. 8).

### GRADE evaluation on quality of evidence

According to the GRADE framework, the quality of evidence was deemed ‘very low’ across all comparative outcomes. The overall ranking of interventions for mortality was assessed as ‘low’ in quality, whereas, for the remaining three outcomes, it was marked as ‘very low’ (Supplementary Table 4a-4d).

### Sensitivity analyses and subgroup analyses

Sensitivity analyses (Supplementary Fig. 5a-5d) was conducted by excluding two trials that investigated NMES in patients undergoing prolonged MV. The results were largely consistent with the results of the network meta-analysis across the four outcomes, affirming their stability. The application of NMES to various muscle groups across the included trials could have influenced its efficacy. However, a subgroup analysis concerning different muscle groups could not be undertaken due to insufficient data.

## Discussion

To our knowledge, this is the first network meta-analysis examining the efficacy of NMES in critically ill, mechanically ventilated adult patients. We incorporated data from 23 RCTs involving 1312 patients in the quantitative analysis. The study revealed that NMES, both alone and in combination with physical therapy, increased the success rate of extubation when compared to standard ICU care. A combination of NMES and physical therapy showed a higher success rate than NMES used independently. However, no significant improvements in ICU LOS, ventilation duration, or mortality rates were observed when NMES was compared with physical therapy or usual care.

NMES is widely recognized as a rehabilitation tool to restore muscle mass and function in individuals with prolonged immobilization or limited activity, including those with spinal cord injuries, stroke, chronic heart failure, and severe chronic obstructive pulmonary disease (COPD) [48]. Owing to its ability to function without patient cooperation and its utilization of automated equipment, NMES presents as a promising option for critically ill patients. The seminal randomized study by Christina Routsi in 2010 revealed that 55-minute daily sessions of NMES could prevent critical illness polyneuropathy and reduce mechanical ventilation duration compared to routine ICU care [49]. Following this, numerous trials have adopted NMES in ICU settings, particularly in mechanically ventilated patients. Evidence indicated that NMES enhanced muscle strength in COPD patients receiving mechanical ventilation, decreased the incidence of ICU-acquired weakness, and shortened both mechanical ventilation duration and ICU stays [39]. Although NMES may benefits ventilated patients, its impact on clinical outcomes remains contentious, and it is unclear whether NMES is a viable substitute for physical rehabilitation in this population. Our study found that NMES alone was only associated with improved extubation success rate compared with usual ICU care, with no notable advancements in ICU length of stay, mechanical ventilation duration, or mortality compared to physical therapy. Furthermore, physical therapy surpassed NMES in improving these outcomes as it showed better hierarchy rankings. Our finding is consistent with another network meta-analysis which included 43 RCTs and investigated all types of rehabilitation interventions in adult critically ill patients and found greater benefits from individualized physical therapy over NMES in reducing ventilation time and ICU stays [50]. It appears that NMES alone is insufficient for enhancing outcomes in ventilated patients and should not replace physical rehabilitation. Evidence-based recommendations propose that NMES be applied particularly in the initial rehabilitation phase when voluntary muscle contractions are not feasible [10].

Although our analysis indicated that NMES combined with physical therapy ranked best for positively affecting all four measured outcomes, neither combination therapy nor NMES alone demonstrated a positive treatment effects on ICU stay, mechanical ventilation duration, or mortality. These findings are consistent with Guillaume Fossat [51], who noted no improvements in muscle strength or ventilation duration when electrical stimulation was combined with early rehabilitation in a sizable ICU study. This lack of outcomes improvement may be attributable to inadequate intervention durations in the included trials, most of which spanned approximately 2 weeks, with the shortest being 7 days. It has been demonstrated that substantial rehabilitation benefits accrue in patients with prolonged ICU stays who receive sufficient intervention dosages. Shorter interventions cannot be compensated for by increased frequency, early initiation, or a higher daily dose of exercise [11].In a prospective RCT trail, Gondin et al. demonstrated that NMES could elicit morphological changes in the muscle, but only for programs longer than 4 weeks in healthy man [52].For optimized outcomes, longer rehabilitation interventions may be necessary for ventilated patients.

Currently, physical rehabilitation remains the only intervention with proven benefits for critically ill patients [53]. The superior ranking of NMES+PT in our study suggests that combining NMES with physical rehabilitation could be a more effective rehabilitative approach for ventilated patients. Future studies should investigate the impact of this combined therapy.

Notably, the included studies in our analysis applied NMES to various muscle groups using different stimulation parameters (frequency, pulse width, intensity), and there is no consensus on optimal settings. With reference to the available data, it is suggested that NMES be executed for durations ranging from 25 to 60 minutes daily. The advised approach prescribes the utilization of a wide pulse and high frequency; specifically for the quadriceps muscle, a frequency of 45 Hz combined with a pulse width of 375 μs is recommended. The stimulation intensity should cause visible muscular contractions, ideally set at a minimum intensity of 50 mA but should not surpass 100 mA [4, 54, 55]. In our study, the majority of the trials featured stimulation parameters that were closely aligned with these recommendations, predominantly opting for a frequency of 50 Hz and a pulse width of 400 μs to induce visible muscular contractions. Preliminary evidence suggests that the parameters applied by most trials were adequate to elicit an enhancement in muscular strength. However, the determination of superior parameters is still unresolved, highlighting the need for further investigation to identify the optimal NMES settings for this specific patient demographic.

Regarding safety, included studies deemed NMES a well-tolerated and safe intervention, with no reports of severe or life-threatening adverse events and only minor complications such as prickling sensations [29]. Most intervention sessions were completed, with only a few stopped prematurely due to NMES intervention.

### Limitation

This study has several limitations. Firstly, as a network meta-analysis, it incorporates a relatively small number of studies, with many of these being single-center trials characterized by limited sample sizes. Such constraints may have resulted in insufficient statistical power to detect differences in intervention effects on clinical outcomes. Secondly, this study lacks outcome parameters for assessing muscle quantity and quality, such as muscle thickness, Medical Research Council Sum Score (MRCs), and functional outcomes. This is due to the high degree of heterogeneity in the methods used to measure these parameters, and the available data were inadequate for a network analysis. Thirdly, the majority of the included studies exhibited a medium to high risk of bias; consequently, the quality of the evidence for all comparisons across the four outcomes was considered very low, as evaluated using the GRADE approach. This underscores the need for further well-controlled trials.

## Conclusion

In conclusion, NMES presents as a feasible and safe intervention for critically ill patients undergoing mechanical ventilation. Both standalone NMES and NMES combined with physical therapy have demonstrated improvements in extubation success rate compared with usual ICU care. Moreover, the incorporation of NMES with physical therapy has demonstrated an enhanced extubation success rate, distinctly superior to NMES used alone. Furthermore, the combination of NMES with physiotherapy showed a better ranking over PT or NMES alone in improving clinical outcomes such as ICU LOS, ventilation duration, extubation success rate, and mortality in this population. However, the quality of evidence remains low to very low, due to concerns of bias and imprecision. Therefore, future RCTs with larger sample sizes and more rigorous methodological designs are necessary.

### Supplementary Information

**Additional file 1:.** Supplementary Tables and Figures

## Data Availability

All data generated or analysed during this study are included in this published article (and its supplementary files).

## Abbreviations

ICU: Intensive care unit
MV: Mechanical ventilation
LOS: Length of stay
NMES: Neuromuscular electrical stimulation
PT: Physical therapy
NMES+PT: NMES combined with PT
CG: Usual
ICU: Care
APACHE II: Acute Physiology and Chronic Health Evaluation II
BMI: Body mass index
COPD: Chronic obstructive pulmonary disease
SUCRA: Surface under the cumulative ranking curve
PRISMA-NMA: Preferred Reporting Items for Systematic Reviews and Meta-Analyses for Network Meta-Analyses
ORs: Odds ratios
CIs: Confidence intervals
MDs: Mean differences
SD: Standard deviation
GRADE: Grading of Recommendations Assessment, Development and Evaluation
