# Supplementary material for: Effect of neuromuscular electrical stimulation in critically ill adults with mechanical ventilation: a systematic review and network meta-analysis
Source: BMC Pulm Med. 2024 Jan 25;24:56. doi: 10.1186/s12890-024-02854-9 (PMC10811936; doi:10.1186/s12890-024-02854-9)
Supplement: Supplementary file 1 — Additional file 1:. Supplementary Tables and Figures [file 12890_2024_2854_MOESM1_ESM.doc]

**Supplementary Table 1:** **PRISMA NMA Checklist**

| **Section/Topic** | **Item #** | **Checklist Item** | **Reported on Page #** |
| --- | --- | --- | --- |
| **TITLE** |  |  |  |
| Title | 1 | Identify the report as a systematic review *incorporating a network meta-analysis (or related form of meta-analysis).* | 1 |
|  |  |  |  |
| **ABSTRACT** |  |  |  |
| Structured summary | 2 | Provide a structured summary including, as applicable:  **Background:** main objectives  **Methods:** data sources; study eligibility criteria, participants, and interventions; study appraisal; and *synthesis methods, such as network meta-analysis.*  **Results:** number of studies and participants identified; summary estimates with corresponding confidence/credible intervals; *treatment rankings may also be discussed. Authors may choose to summarize pairwise comparisons against a chosen treatment included in their analyses for brevity.*  **Discussion/Conclusions:** limitations; conclusions and implications of findings.  **Other:** primary source of funding; systematic review registration number with registry name. | 1-2 |
|  |  |  |  |
| **INTRODUCTION** |  |  |  |
| Rationale | 3 | Describe the rationale for the review in the context of what is already known*, including mention of why a network meta-analysis has been conducted.* | 2-4 |
| Objectives | 4 | Provide an explicit statement of questions being addressed, with reference to participants, interventions, comparisons, outcomes, and study design (PICOS). | 4 |
|  |  |  |  |
| **METHODS** |  |  |  |
| Protocol and registration | 5 | Indicate whether a review protocol exists and if and where it can be accessed (e.g., Web address); and, if available, provide registration information, including registration number. | NR |
| Eligibility criteria | 6 | Specify study characteristics (e.g., PICOS, length of follow-up) and report characteristics (e.g., years considered, language, publication status) used as criteria for eligibility, giving rationale. *Clearly describe eligible treatments included in the treatment network, and note whether any have been clustered or merged into the same node (with justification).* | ***5*** |
| Information sources | 7 | Describe all information sources (e.g., databases with dates of coverage, contact with study authors to identify additional studies) in the search and date last searched. | 5 |
| Search | 8 | Present full electronic search strategy for at least one database, including any limits used, such that it could be repeated. | 5, Supplementary Table 2 |
| Study selection | 9 | State the process for selecting studies (i.e., screening, eligibility, included in systematic review, and, if applicable, included in the meta-analysis). | 6 |
| Data collection process | 10 | Describe method of data extraction from reports (e.g., piloted forms, independently, in duplicate) and any processes for obtaining and confirming data from investigators. | 6 |
| Data items | 11 | List and define all variables for which data were sought (e.g., PICOS, funding sources) and any assumptions and simplifications made. | 6 |
| **Geometry of the network** | **S1** | Describe methods used to explore the geometry of the treatment network under study and potential biases related to it. This should include how the evidence base has been graphically summarized for presentation, and what characteristics were compiled and used to describe the evidence base to readers. | 7-8 |
| Risk of bias within individual studies | 12 | Describe methods used for assessing risk of bias of individual studies (including specification of whether this was done at the study or outcome level), and how this information is to be used in any data synthesis. | 6-7 |
| Summary measures | 13 | State the principal summary measures (e.g., risk ratio, difference in means). *Also describe the use of additional summary measures assessed, such as treatment rankings and surface under the cumulative ranking curve (SUCRA) values, as well as modified approaches used to present summary findings from meta-analyses.* | 7-8 |
| Planned methods of analysis | 14 | Describe the methods of handling data and combining results of studies for each network meta-analysis. This should include, but not be limited to:   - *Handling of multi-arm trials;* - *Selection of variance structure;* - *Selection of prior distributions in Bayesian analyses; and* - *Assessment of model fit.* | 7-8 |
| **Assessment of Inconsistency** | **S2** | Describe the statistical methods used to evaluate the agreement of direct and indirect evidence in the treatment network(s) studied. Describe efforts taken to address its presence when found. | 7-8 |
| Risk of bias across studies | 15 | Specify any assessment of risk of bias that may affect the cumulative evidence (e.g., publication bias, selective reporting within studies). | 8 |
| Additional analyses | 16 | Describe methods of additional analyses if done, indicating which were pre-specified. This may include, but not be limited to, the following:   - Sensitivity or subgroup analyses; - Meta-regression analyses; - *Alternative formulations of the treatment network; and* - *Use of alternative prior distributions for Bayesian analyses (if applicable).* | 8 |
|  |  |  |  |
| **RESULTS†** |  |  |  |
| Study selection | 17 | Give numbers of studies screened, assessed for eligibility, and included in the review, with reasons for exclusions at each stage, ideally with a flow diagram. | 8 |
| **Presentation of network structure** | **S3** | Provide a network graph of the included studies to enable visualization of the geometry of the treatment network. | 8, Fig. 2 |
| **Summary of network geometry** | **S4** | Provide a brief overview of characteristics of the treatment network. This may include commentary on the abundance of trials and randomized patients for the different interventions and pairwise comparisons in the network, gaps of evidence in the treatment network, and potential biases reflected by the network structure. | 8-9, Fig. 2 |
| Study characteristics | 18 | For each study, present characteristics for which data were extracted (e.g., study size, PICOS, follow-up period) and provide the citations. | 8-9, Table 1 |
| Risk of bias within studies | 19 | Present data on risk of bias of each study and, if available, any outcome level assessment. | 10, Fig. 3, Supplementary Table 3 |
| Results of individual studies | 20 | For all outcomes considered (benefits or harms), present, for each study: 1) simple summary data for each intervention group, and 2) effect estimates and confidence intervals. *Modified approaches may be needed to deal with information from larger networks.* | 10 |
| Synthesis of results | 21 | Present results of each meta-analysis done, including confidence/credible intervals. *In larger networks, authors may focus on comparisons versus a particular comparator (e.g. placebo or standard care), with full findings presented in an appendix. League tables and forest plots may be considered to summarize pairwise comparisons.* If additional summary measures were explored (such as treatment rankings), these should also be presented. | 10-12 |
| **Exploration for inconsistency** | **S5** | Describe results from investigations of inconsistency. This may include such information as measures of model fit to compare consistency and inconsistency models, *P* values from statistical tests, or summary of inconsistency estimates from different parts of the treatment network. | 11-12, Supplementary Fig. 2a-2d |
| Risk of bias across studies | 22 | Present results of any assessment of risk of bias across studies for the evidence base being studied. | 12-13, Supplementary Table 4a-4d |
| Results of additional analyses | 23 | Give results of additional analyses, if done (e.g., sensitivity or subgroup analyses, meta-regression analyses, alternative network geometries studied, alternative choice of prior distributions for Bayesian analyses, and so forth). | 13, Supplementary Fig. 5a-5d |
|  |  |  |  |
| **DISCUSSION** |  |  |  |
| Summary of evidence | 24 | Summarize the main findings, including the strength of evidence for each main outcome; consider their relevance to key groups (e.g., healthcare providers, users, and policy-makers). | 13 |
| Limitations | 25 | Discuss limitations at study and outcome level (e.g., risk of bias), and at review level (e.g., incomplete retrieval of identified research, reporting bias). *Comment on the validity of the assumptions, such as transitivity and consistency. Comment on any concerns regarding network geometry (e.g., avoidance of certain comparisons).* | 16 |
| Conclusions | 26 | Provide a general interpretation of the results in the context of other evidence, and implications for future research. | 17 |
|  |  |  |  |
| **FUNDING** |  |  |  |
| Funding | 27 | Describe sources of funding for the systematic review and other support (e.g., supply of data); role of funders for the systematic review. This should also include information regarding whether funding has been received from manufacturers of treatments in the network and/or whether some of the authors are content experts with professional conflicts of interest that could affect use of treatments in the network. | 17 |

PICOS = population, intervention, comparators, outcomes, study design.

* Text in italics indicateS wording specific to reporting of network meta-analyses that has been added to guidance from the PRISMA statement.

† Authors may wish to plan for use of appendices to present all relevant information in full detail for items in this section.

**Supplementary Table 2: Search strategy**

|  | **Search strategy（Pubmed）** | **Results** |
| --- | --- | --- |
| #1 | ("Respiration, Artificial"[Mesh]) OR ((((((((Artificial Respiration[Title/Abstract]) OR (Artificial Respirations[Title/Abstract])) OR (Respirations, Artificial[Title/Abstract])) OR (Ventilation, Mechanical[Title/Abstract])) OR (Mechanical Ventilations[Title/Abstract])) OR (Ventilations, Mechanical[Title/Abstract])) OR (Mechanical Ventilation[Title/Abstract])) OR ((mechanically ventilated[Title/Abstract]))) | 129,975 |
| #2 | (randomized controlled trial[Publication Type] OR controlled clinical trial[Publication Type] OR randomized[Title/Abstract] OR placebo[Title/Abstract] OR clinical trials as topic[MeSH Terms:noexp] OR randomly[Title/Abstract] OR trial[Title]) NOT (animals[MeSH Terms] NOT (humans[MeSH Terms] AND animals[MeSH Terms])) | 1,457,951 |
| #3 | (((((("Electric Stimulation"[Mesh]) OR (Electrical Stimulations[Title/Abstract])) OR (Stimulation, Electrical[Title/Abstract])) OR (Stimulations, Electrical[Title/Abstract])) OR (Stimulation, Electric[Title/Abstract])) OR (((((Electric Stimulations[Title/Abstract]) OR (Stimulations, Electric[Title/Abstract])) OR (electrical diaphragmatic stimulation[Title/Abstract])) OR (nerve stimulation[Title/Abstract])) OR (Electrical muscle stimulation[Title/Abstract]))) OR ((("Electric Stimulation Therapy"[Mesh]) OR (((Therapeutic Electrical Stimulation[Title/Abstract]) OR (Electrical Stimulation, Therapeutic[Title/Abstract])) OR (Stimulation, Therapeutic Electrical[Title/Abstract]))) OR (((((((((((Therapeutic Electric Stimulation[Title/Abstract]) OR (Electric Stimulation, Therapeutic[Title/Abstract])) OR (Stimulation, Therapeutic Electric[Title/Abstract])) OR (Electrical Stimulation Therapy[Title/Abstract])) OR (Stimulation Therapy, Electrical[Title/Abstract])) OR (Therapy, Electrical Stimulation[Title/Abstract])) OR (Therapy, Electric Stimulation[Title/Abstract])) OR (Stimulation Therapy, Electric[Title/Abstract])) OR (Electrotherapy[Title/Abstract])) OR (Electrotherapy, Interferential Current[Title/Abstract])) OR (Interferential Current Electrotherapy[Title/Abstract]))) | 233,758 |
| #4 | #1 AND #2 AND #3 | 146 |
|  | **Search strategy（Embase）** | **Results** |
| #1 | 'artificial ventilation'/exp OR 'artificial ventilation' | 302,948 |
| #2 | 'mechanical ventilation*':ab,ti OR 'artificial respiration*':ab,ti OR 'respiration*, artificial':ab,ti OR 'ventilation*, mechanical':ab,ti | 99,036 |
| #3 | #1 OR #2 | 314,929 |
| #4 | 'electrostimulation'/exp OR 'electrostimulation' | 96,703 |
| #5 | 'electrical stimulation':ab,ti OR 'electrical stimulations':ab,ti OR 'stimulation, electrical':ab,ti OR 'stimulations, electrical':ab,ti OR 'electrostimulation':ab,ti OR 'electric stimulations':ab,ti OR 'stimulations, electric':ab,ti OR 'electric stimulation therapy':ab,ti OR 'therapeutic electrical stimulation':ab,ti OR 'electrical stimulation, therapeutic':ab,ti OR 'stimulation, therapeutic electrical':ab,ti OR 'therapeutic electric stimulation':ab,ti OR 'electric stimulation, therapeutic':ab,ti OR 'stimulation, therapeutic electric':ab,ti OR 'electrical stimulation therapy':ab,ti OR 'stimulation therapy, electrical':ab,ti OR 'therapy, electrical stimulation':ab,ti OR 'therapy, electric stimulation':ab,ti OR 'stimulation therapy, electric':ab,ti OR 'electrotherapy':ab,ti OR 'electrotherapy, interferential current':ab,ti OR 'interferential current electrotherapy':ab,ti OR 'electrical diaphragmatic stimulation':ab,ti OR 'nerve stimulation':ab,ti OR 'electrical muscle stimulation':ab,ti | 106,151 |
| #6 | #4 OR #5 | 156,134 |
| #7 | 'trial':ti | 418,523 |
| #8 | 'clinical trial (topic)'/exp | 451,488 |
| #9 | 'random':ab,ti OR 'placebo':ab,ti OR 'double-blind':ab,ti OR 'randomized':ab,ti OR 'randomly':ab,ti | 2,017,788 |
| #10 | #7 OR #8 OR #9 | 2,448,663 |
| #11 | #3 AND #6 AND #10 | 265 |
|  | **Search strategy（Corchrane）** | Results |
| #1 | (mechanical ventilation):ti,ab,kw OR (Artificial Respiration):ti,ab,kw OR (Respiration, Artificial):ti,ab,kw OR (Ventilation, Mechanical):ti,ab,kw OR (Ventilation, Mechanical):ti,ab,kw (Word variations have been searched) | 19923 |
| #2 | (mechanically ventilated):ti,ab,kw (Word variations have been searched) | 17819 |
| #3 | MeSH descriptor: [Respiration, Artificial] explode all trees | 8350 |
| #4 | #1 OR #2 OR #3 | 22756 |
| #5 | MeSH descriptor: [Electric Stimulation] explode all trees | 2305 |
| #6 | (Electrical Stimulation):ti,ab,kw OR (Stimulation, Electrical):ti,ab,kw OR (Stimulation, Electric):ti,ab,kw OR (nerve stimulation):ti,ab,kw OR (electrical diaphragmatic stimulation):ti,ab,kw (Word variations have been searched) | 22645 |
| #7 | (Electrical muscle stimulation):ti,ab,kw OR (Therapeutic Electrical Stimulation):ti,ab,kw OR (Electrical Stimulation, Therapeutic):ti,ab,kw OR (Stimulation, Therapeutic Electrical):ti,ab,kw OR (Therapeutic Electric Stimulation):ti,ab,kw (Word variations have been searched) | 6727 |
| #8 | (Electric Stimulation, Therapeutic):ti,ab,kw OR (Stimulation, Therapeutic Electric):ti,ab,kw OR (Stimulation Therapy, Electrical):ti,ab,kw OR (Therapy, Electrical Stimulation):ti,ab,kw OR (Therapy, Electric Stimulation):ti,ab,kw (Word variations have been searched) | 8626 |
| #9 | (Stimulation Therapy, Electric):ti,ab,kw OR (Electrotherapy):ti,ab,kw (Word variations have been searched) | 8798 |
| #10 | MeSH descriptor: [Electric Stimulation Therapy] explode all trees | 10182 |
| #11 | #5 OR #6 OR #7 OR #8 OR #9 OR #10 | 28952 |
| #12 | #4 AND #11 | 372 |
|  | **Search strategy（Web of science）** |  |
| #1 | ((((((TS=("clinical trial*" )) OR TS=("double-blind")) OR TS=(placebo)) OR TS=(random*)) OR TS=("controlled clinical trial")) OR TS=("randomized controlled trial*")) OR TS=(RCT) and Preprint Citation Index | 4256361 |
| #2 | ((((((((((((((((TS=("Electric* Stimulation*")) OR TS=("Stimulation*, Electric*")) OR TS=("electric* diaphragmatic stimulation*")) OR TS=("Stimulation* Therapy, Electric*")) OR TS=(Electrotherapy)) OR TS=("Electrotherapy, Interferential Current")) OR TS=("Interferential Current Electrotherapy“)) OR TS=("Stimulation*, Therapeutic Electric*")) OR TS=("nerve stimulation*")) OR TS=("Electric* muscle stimulation*")) OR TS=("Neuromuscular electrical stimulation*")) OR TS=("functional electric stimulation*" )) OR TS=("transcutaneous electrical nerve stimulation*")) OR TS=(PNS)) OR TS=(NMES)) OR TS=(TEDS)) OR TS=(FES) and Preprint Citation Index | 278767 |
| #3 | ((((TS=("artificial Respiration*")) OR TS=("Respiration*, Artificial")) OR TS=("Ventilation*, Mechanical")) OR TS=("Mechanical Ventilation*")) OR TS=("mechanically ventilated") and Preprint Citation Index | 136651 |
| #4 | #1 AND #2 AND #3 | 214 |
|  | **Search strategy（PEDro）** |  |
| #1 | ((Abstract & Title=("mechanically ventilated*") AND Therapy= (electrotherapies,heat,cole) )AND Method= (clinical trail) | 9 |
| #2 | ((Abstract & Title=("Mechanical Ventilation*") AND Therapy= (electrotherapies,heat,cole) )AND Method= (clinical trail) | 21 |
| #3 | ((Abstract & Title=("Ventilation, Mechanical") AND Therapy= (electrotherapies,heat,cole) )AND Method= (clinical trail) | 0 |
| #4 | ((Abstract & Title=("Ventilat*") AND Therapy= (electrotherapies,heat,cole) )AND Method= (clinical trail) | 44 |
| #4 | #1 OR #2 OR #3 OR #4 | 51 |

**Supplementary Table 3: Results of risk assessment of bias in the included studies**

| **Study** | **random sequence generation** | **allocation concealment** | **blinding of participants** | **blinding of outcome assessment** | **incomplete outcome data** | **selective reporting** | **other biases** |
| --- | --- | --- | --- | --- | --- | --- | --- |
| McCaughey EJ, et al. 2019 | Unclear | Unclear | Low | Low | Low | Unclear | Low |
| Jonkman AH, et al. 2020 | Unclear | Unclear | Low | Low | Low | Unclear | Low |
| Abu-Khaber HA, et al. 2013 | High | Unclear | High | Low | Low | Unclear | Unclear |
| Koutsioumpa E, et al. 2018 | Unclear | Unclear | High | Unclear | Low | Unclear | Unclear |
| Hsin YF, et al. 2022 | Low | Low | High | Unclear | Low | Low | Low |
| Chen YH, et al. 2019 | Low | Low | Low | Low | Low | Low | Low |
| Leite MA, et al.2018 | High | Unclear | High | Unclear | Low | Low | Low |
| Shen SY, et al. 2017 | Unclear | Unclear | High | Unclear | High | Low | Low |
| Dall' Acqua AM, et al. 2017 | Low | Low | Low | Low | Low | Low | Low |
| Kho ME, et al.2015 | Low | Low | Low | Low | Low | Low | Low |
| Dos Santos FV, et al.2020 | Low | Low | Low | Low | Low | Low | Low |
| Silva PE, et al.2019 | Low | Low | High | Low | Low | Low | Low |
| Mahran GSK, et al. 2023 | Low | Low | Low | Low | Unclear | Low | Low |
| Chen S, et al.2019 | Low | Unclear | High | Unclear | Low | Unclear | Low |
| Peng, Lu, et al.2022 | Low | Unclear | High | Unclear | Low | Unclear | Low |
| Verceles AC, et al.2023 | Unclear | Unclear | High | Unclear | Low | Unclear | Low |
| Medrinal C,et al.2023 | Low | Low | Low | Low | Low | Low | Low |
| Liu Y, et al.2023 | Low | Unclear | High | Unclear | Low | Low | Low |
| Campos DR, et al.2022 | Low | Low | High | High | Low | Low | Low |
| Othman SY, et al.2023 | Low | Low | High | Low | Low | Low | Unclear |
| Olimpio JH, et al.2023 | Low | Unclear | High | Low | Low | Low | Low |
| Nakanishi N, et al.2020 | Low | Low | High | Low | Low | Low | Low |
| Vieira L, et al.2023 | Low | Low | High | Low | Low | Low | Low |

**Supplementary Fig. 1a** The contribution plot for ICU LOS. The size of the circle is proportional to the percentage contribution of the column-defining direct comparison to the row-defining network estimate. A=NMES, B=PT, C=NMES+PT, D=CG.

**Supplementary Fig. 1b** The contribution plot for ventilation duration. The size of the circle is proportional to the percentage contribution of the column-defining direct comparison to the row-defining network estimate. A=NMES, B=PT, C=NMES+PT, D=CG.

**Supplementary Fig. 1c** The contribution plot for extubation success rate. The size of the circle is proportional to the percentage contribution of the column-defining direct comparison to the row-defining network estimate. A=NMES, B=PT, C=NMES+PT, D=CG.

**Supplementary Fig. 1d** The contribution plot for mortality. The size of the circle is proportional to the percentage contribution of the column-defining direct comparison to the row-defining network estimate. A=NMES, B=PT, C=NMES+PT, D=CG.

**Supplementary Fig. 2a** Forest plot of inconsistence check for all closed loops in network for ICU LOS. A=NMES, B=PT, C=NMES+PT, D=CG.

**Supplementary Fig. 2b** Forest plot of inconsistence check for all closed loops in network for ventilation duration.

**Supplementary Fig. 2c** Forest plot of inconsistence check for all closed loops in network for extubation success rate.

**Supplementary Fig. 2d** Forest plot of inconsistence check for all closed loops in network for mortality. A=NMES, B=PT, C=NMES+PT, D=CG.

**Supplementary Fig. 3a** Evaluation of the inconsistency by node-splitting model for ICU LOS. A=NMES, B=PT, C=NMES+PT, D=CG.

**Supplementary Fig. 3b** Evaluation of the inconsistency by node-splitting model for ventilation duration. A=NMES, B=PT, C=NMES+PT, D=CG.

**Supplementary Fig. 3c** Evaluation of the inconsistency by node-splitting model for extubation success rate. A=NMES, B=PT, C=NMES+PT, D=CG.

**Supplementary Fig. 3d** Evaluation of the inconsistency by node-splitting model for mortality. A=NMES, B=PT, C=NMES+PT, D=CG.

**Supplementary Fig. 4a** Comparison-adjusted funnel plot for ICU LOS.

**Supplementary Fig. 4b** Comparison-adjusted funnel plot for ventilation duration.

**Supplementary Fig. 4c** Comparison-adjusted funnel plot for extubation success rate.

**Supplementary Fig. 4d** Comparison-adjusted funnel plot for mortality.

**Supplementary Table 4-1 Evaluation of the quality of evidence using GRADE framework for ICU LOS**

| **Comparison** | **Study limitation** | **Imprecision** | **Inconsistency** | **Indirectness** | **Publication bias** | **Confidence** |
| --- | --- | --- | --- | --- | --- | --- |
| NMES VS PT | 86.9% of the estimate from studies at high risk, 13.1% at moderate risk. | WMD=0.40, 95%CI:-7.47 to 8.27 | No inconsistency between direct and indirect estimate. | Most comparisons include few studies. | The funnel plot for the direct comparison is not suggestive of any dominant publication bias. | Very Low (Downgrade by four levels due to study limitation (for two levels), imprecision and indirectness. |
| NMES vs NMES+PT | 80.1% of the estimate from studies at high risk, 19.9% at moderate risk. | WMD=2.89, 95%CI:-4.988 to 10.77 | No inconsistency between direct and indirect estimate. | Most comparisons include few studies. | The funnel plot for the direct comparison is not suggestive of any dominant publication bias. | Very Low (Downgrade by four levels due to study limitation (for two levels), imprecision and indirectness. |
| NMES vs CG | 60.1% of the estimate from studies at high risk, 39.9% at moderate risk. | WMD=-2.05, 95%CI:-8.84 to 4.73 | No inconsistency between direct and indirect estimate. | Most comparisons include few studies. | The funnel plot for the direct comparison is not suggestive of any dominant publication bias. | Very Low (Downgrade by four levels due to study limitation(for two levels), imprecision and indirectness. |
| NMES+PT vs PT | 95.1% of the estimate from studies at high risk, 4.9% at moderate risk. | WMD=-2.49, 95%CI: -6.90 to 1.91 | No inconsistency between direct and indirect estimate. | Most comparisons include few studies. | The funnel plot for the direct comparison is not suggestive of any dominant publication bias. | Very Low (Downgrade by four levels due to study limitation (for two levels), imprecision and indirectness. |
| PT vs CG | 78.8% of the estimate from studies at high risk, 21.2% at moderate risk. | WMD=-2.45  , 95%CI: −11.04 to 6.14 | No inconsistency between direct and indirect estimate. | Most comparisons include few studies. | The funnel plot for the direct comparison is not suggestive of any dominant publication bias. | Very Low (Downgrade by four levels due to study limitation(for two levels), imprecision and indirectness. |
| NMES+PT vs CG | 78.2% of the estimate from studies at high risk, 21.8% at moderate risk. | WMD=-4.94, 95%CI: -13.54 to 3.65 | No inconsistency between direct and indirect estimate. | Most comparisons include few studies. | The funnel plot for the direct comparison is not suggestive of any dominant publication bias. | Very Low (Downgrade by four levels due to study limitation(for two levels), imprecision and indirectness. |
| Ranking of treatment | 80.4% of the estimate from studies at high risk, 19.6% at moderate risk. | SUCRA plots suggested precision in a ranking of treatments. | No significant inconsistency in test of global inconsistency (P = 0.9844, and no significant in local inconsistency. | Most comparisons include few studies. | The comparison-adjusted funnel plot for the network is not suggestive of any dominant publication bias. | Very Low (Downgrade by three levels due to study limitation(for two levels) and indirectness. |

**Table 4-2 Evaluation of the quality of evidence using GRADE framework for ventilation duration**

| **Comparison** | **Study limitation** | **Imprecision** | **Inconsistency** | **Indirectness** | **Publication bias** | **Confidence** |
| --- | --- | --- | --- | --- | --- | --- |
| NMES VS PT | 100% of the estimate from studies at high risk | WMD=0.62, 95%CI:-7.83 to 9.06 | No inconsistency between direct and indirect estimate. | Most comparisons include few studies. | The funnel plot for the direct comparison is not suggestive of any dominant publication bias. | Very Low (Downgrade by four levels due to study limitation (for two levels), imprecision and indirectness. |
| NMES vs NMES+PT | 100% of the estimate from studies at high risk | WMD=3.70, 95%CI:-4.36to 11.76 | No inconsistency between direct and indirect estimate. | Most comparisons include few studies. | The funnel plot for the direct comparison is not suggestive of any dominant publication bias. | Very Low (Downgrade by four levels due to study limitation (for two levels), imprecision and indirectness. |
| NMES vs CG | 100% of the estimate from studies at high risk | WMD=-2.75, 95%CI:-8.62 to 3.12 | No inconsistency between direct and indirect estimate. | Most comparisons include few studies. | The funnel plot for the direct comparison is not suggestive of any dominant publication bias. | Very Low (Downgrade by four levels due to study limitation(for two levels), imprecision and indirectness. |
| NMES+PT vs PT | 100% of the estimate from studies at high risk | WMD=-3.08, 95%CI: -7.83 to 1.66 | No inconsistency between direct and indirect estimate. | Most comparisons include few studies. | The funnel plot for the direct comparison is not suggestive of any dominant publication bias. | Very Low (Downgrade by four levels due to study limitation(for two levels), imprecision and indirectness. |
| PT vs CG | 100% of the estimate from studies at high risk | WMD=-3.37, 95%CI: -12.36 to 5.62 | No inconsistency between direct and indirect estimate. | Most comparisons include few studies. | The funnel plot for the direct comparison is not suggestive of any dominant publication bias. | Very Low (Downgrade by four levels due to study limitation (for two levels), imprecision and indirectness. |
| NMES+PT vs CG | 100% of the estimate from studies at high risk | WMD=-6.45, 95%CI: -15.04 to 2.14 | No inconsistency between direct and indirect estimate. | Most comparisons include few studies. | The funnel plot for the direct comparison is not suggestive of any dominant publication bias. | Very Low (Downgrade by four levels due to study limitation (for two levels) ,  imprecision and indirectness. |
| Ranking of treatment | 100% of the estimate from studies at high risk | SUCRA plots suggested precision in a ranking of treatments. | No significant inconsistency in test of global inconsistency (P = 0.8107), and no significant in local inconsistency. | Most comparisons include few studies. | The comparison-adjusted funnel plot for the network is not suggestive of any dominant publication bias. | Very Low  (Downgrade by three levels due to study limitation(for two levels) and indirectness. |

**Table 4-3 Evaluation of the quality of evidence using GRADE framework for extubation success rate**

| **Comparison** | **Study limitation** | **Imprecision** | **Inconsistency** | **Indirectness** | **Publication bias** | **Confidence** |
| --- | --- | --- | --- | --- | --- | --- |
| NMES VS PT | 100% of the estimate from studies at high risk. | WMD=0.64, 95%CI:0.24 to 1.73 | No inconsistency between direct and indirect estimate. | Most comparisons include few studies. | The funnel plot for the direct comparison is suggestive of dominant publication bias. | Very Low (Downgrade by five levels due to study limitation (for two levels), imprecision , publication bias and indirectness. |
| NMES vs NMES+PT | 100% of the estimate from studies at high risk. | WMD=0.93, 95%CI:0.11 to 0.93 | No inconsistency between direct and indirect estimate. | Most comparisons include few studies. | The funnel plot for the direct comparison is suggestive of dominant publication bias. | Very Low (Downgrade by four levels due to study limitation (for two levels), publication bias and indirectness. |
| NMES vs CG | 100% of the estimate from studies at high risk. | WMD=1.85, 95%CI:1.11 to 3.08 | No inconsistency between direct and indirect estimate. | Most comparisons include few studies. | The funnel plot for the direct comparison is suggestive of dominant publication bias. | Very Low (Downgrade by four levels due to study limitation (for two levels), publication bias and indirectness. |
| NMES+PT vs PT | 100% of the estimate from studies at high risk. | WMD=2.04, 95%CI: 0.91 to 4.53 | No inconsistency between direct and indirect estimate. | Most comparisons include few studies. | The funnel plot for the direct comparison is suggestive of dominant publication bias. | Very Low (Downgrade by five levels due to study limitation (for two levels), imprecision, publication bias and indirectness. |
| PT vs CG | 100% of the estimate from studies at high risk. | WMD=2.89, 95%CI: 0.95 to 8.84 | No inconsistency between direct and indirect estimate. | Most comparisons include few studies. | The funnel plot for the direct comparison is suggestive of dominant publication bias. | Very Low (Downgrade by five levels due to study limitation (for two levels), imprecision, publication bias and indirectness. |
| NMES+PT vs CG | 100% of the estimate from studies at high risk. | WMD=5.89, 95%CI: 1.77 to 19.65 | No inconsistency between direct and indirect estimate. | Most comparisons include few studies. | The funnel plot for the direct comparison is suggestive of dominant publication bias. | Very Low (Downgrade by four levels due to study limitation (for two levels), publication bias and indirectness. |
| Ranking of treatment | 100% of the estimate from studies at high risk. | SUCRA plots suggested precision in a ranking of treatments. | No significant inconsistency in test of global inconsistency (P = 0.3692), and no significant in local inconsistency. | Most comparisons include few studies. | The funnel plot for the direct comparison is suggestive of dominant publication bias. | Very Low (Downgrade by four levels due to study limitation (for two levels), publication bias and indirectness. |

**Supplementary Table 4-4 Evaluation of the quality of evidence using GRADE framework for mortality**

| **Comparison** | **Study limitation** | **Imprecision** | **Inconsistency** | **Indirectness** | **Publication bias** | **Confidence** |
| --- | --- | --- | --- | --- | --- | --- |
| NMES VS PT | 48.8% of the estimate from studies at high risk, 51.2% at low risk. | WMD=1.29, 95%CI:0.36 to 4.62 | No inconsistency between direct and indirect estimate. | Most comparisons include few studies. | The funnel plot for the direct comparison is not suggestive of any dominant publication bias. | Very Low (Downgrade by three levels due to study limitation, imprecision and indirectness. |
| NMES vs NMES+PT | 49.7% of the estimate from studies at high risk, 50.3% at low risk. | WMD=1.63  , 95%CI:0.45 to 5.89 | No inconsistency between direct and indirect estimate. | Most comparisons include few studies. | The funnel plot for the direct comparison is not suggestive of any dominant publication bias. | Very Low (Downgrade by three levels due to study limitation, imprecision and indirectness. |
| NMES vs CG | 77.3% of the estimate from studies at high risk, 22.7% at low risk. | WMD=0.62, 95%CI:0.32 to 1.20 | No inconsistency between direct and indirect estimate. | Most comparisons include few studies. | The funnel plot for the direct comparison is not suggestive of any dominant publication bias. | Very Low (Downgrade by four levels due to study limitation(for two levels), imprecision and indirectness. |
| NMES+PT vs PT | 89.0% of the estimate from studies at high risk, 11.0% at low risk. | WMD=0.79, 95%CI: 0.52 to 1.21 | No inconsistency between direct and indirect estimate. | Most comparisons include few studies. | The funnel plot for the direct comparison is not suggestive of any dominant publication bias. | Very Low (Downgrade by four levels due to study limitation(for two levels), imprecision and indirectness. |
| PT vs CG | 45.7% of the estimate from studies at high risk, 54.3% at low risk. | WMD=0.48, 95%CI: 0.14 to 1.64 | No inconsistency between direct and indirect estimate. | Most comparisons include few studies. | The funnel plot for the direct comparison is not suggestive of any dominant publication bias. | Very Low (Downgrade by three levels due to study limitation, imprecision and indirectness. |
| NMES+PT vs CG | 46.8% of the estimate from studies at high risk, 53.2% at low risk. | WMD=0.38, 95%CI: 0.11 to 1.31 | No inconsistency between direct and indirect estimate. | Most comparisons include few studies. | The funnel plot for the direct comparison is not suggestive of any dominant publication bias. | Very Low (Downgrade by three levels due to study limitation, imprecision and indirectness. |
| Ranking of treatment | 55.4% of the estimate from studies at high risk, 44.6% at low risk. | SUCRA plots suggested precision in a ranking of treatments. | No significant inconsistency in test of global inconsistency (P = 0.7168), and no significant in local inconsistency. | Most comparisons include few studies. | The comparison-adjusted funnel plot for the network is not suggestive of any dominant publication bias. | Low (Downgrade by two levels due to study limitation and indirectness. |


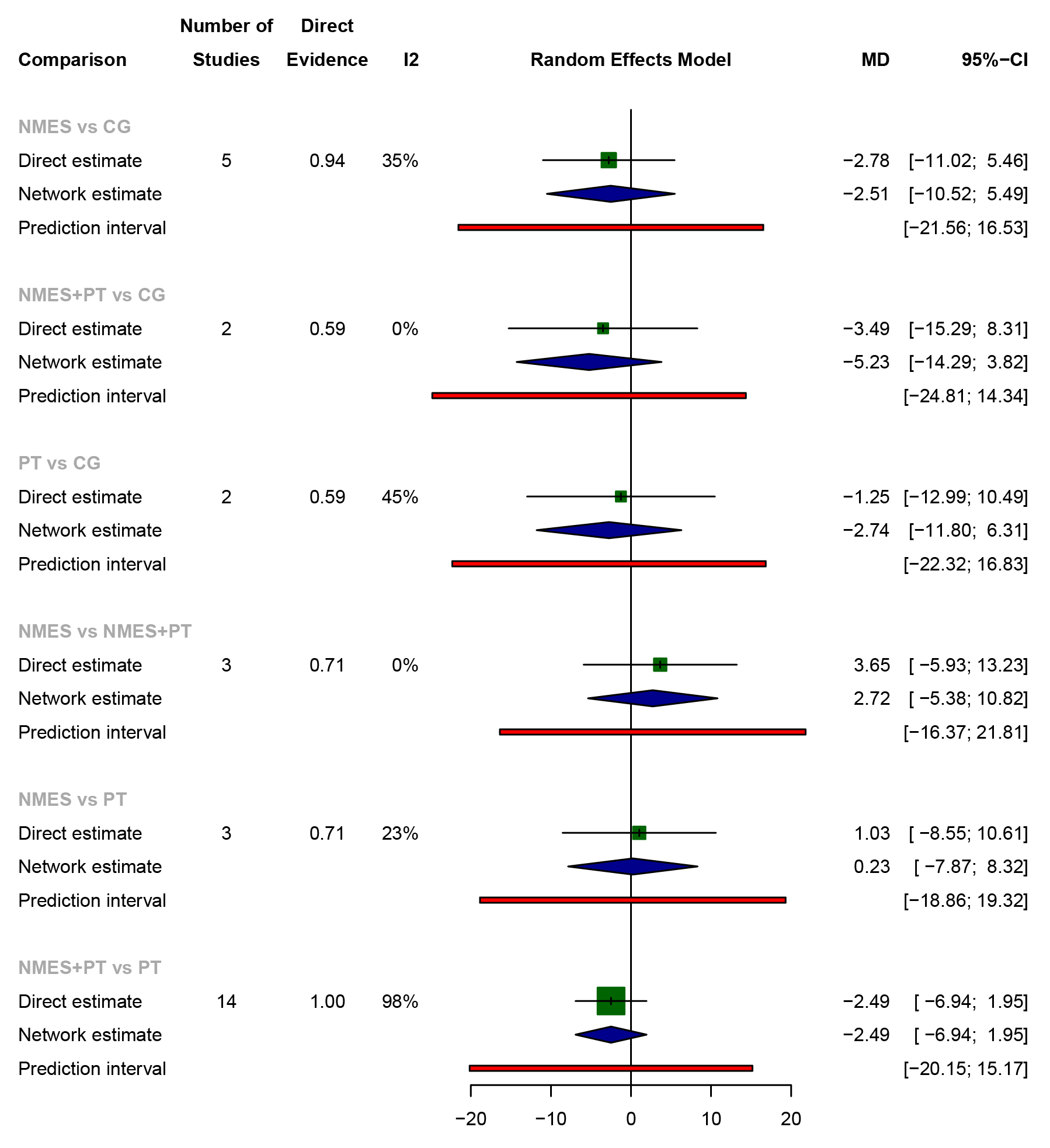


**Supplementary Fig. 5a** Forest plot of pairwise meta-analysis, network meta-analysis and predictive interval for ICU LOS from sensitivity analyses. Both the pairwise meta-analysis and the network meta-analysis revealed no significant differences among the four treatments for ICU LOS. Predictive interval plots suggested no significant heterogeneity in the network meta-analysis among the comparisons for ICU LOS.


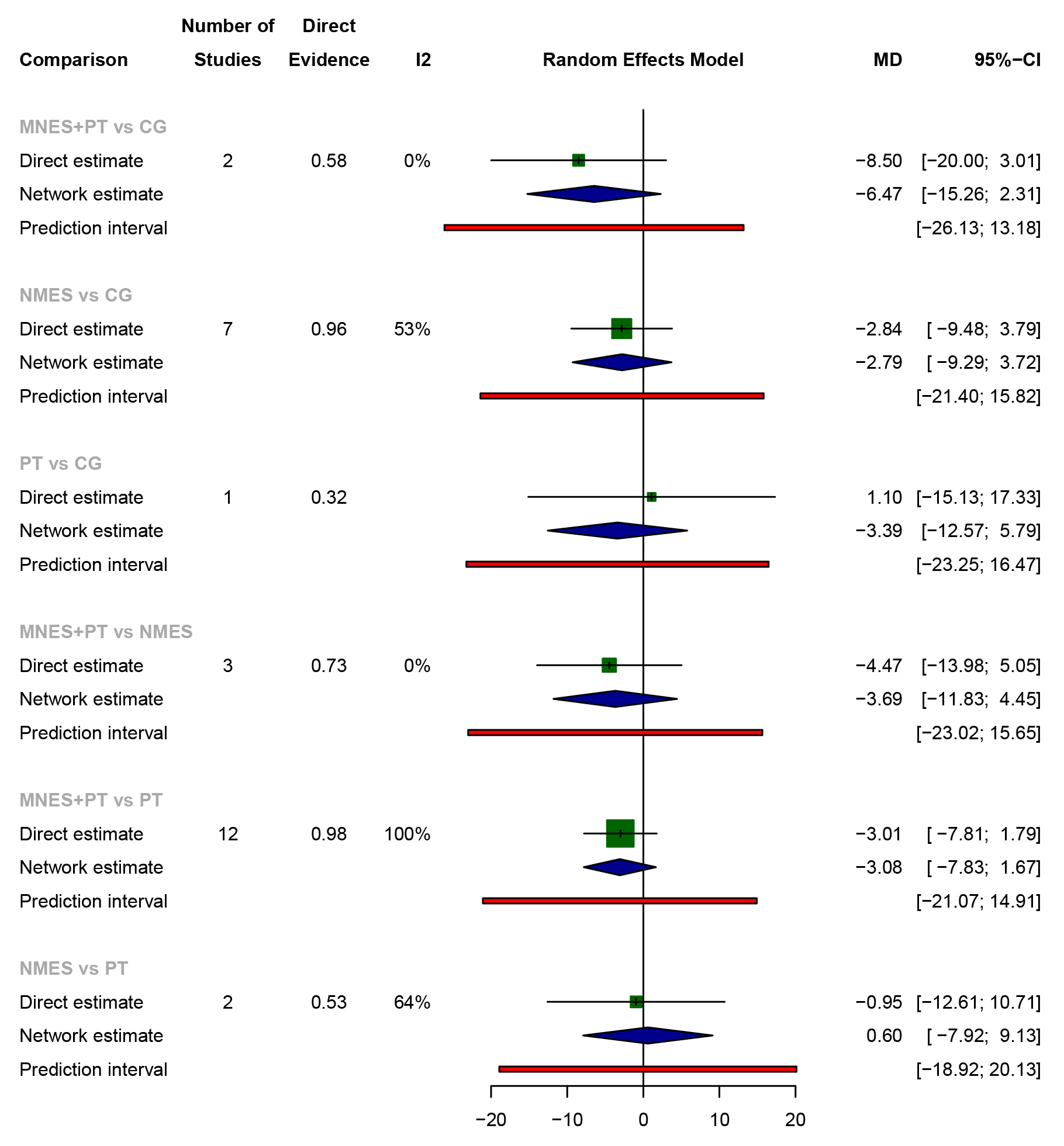


**Supplementary Fig. 5b** Forest plot of pairwise meta-analysis, network meta-analysis and predictive interval for ventilation duration from sensitivity analyses. Both the pairwise meta-analysis and the network meta-analysis revealed no significant differences among the four treatments for ventilation duration. Predictive interval plots suggested no significant heterogeneity in the network meta-analysis among the comparisons for ventilation duration.


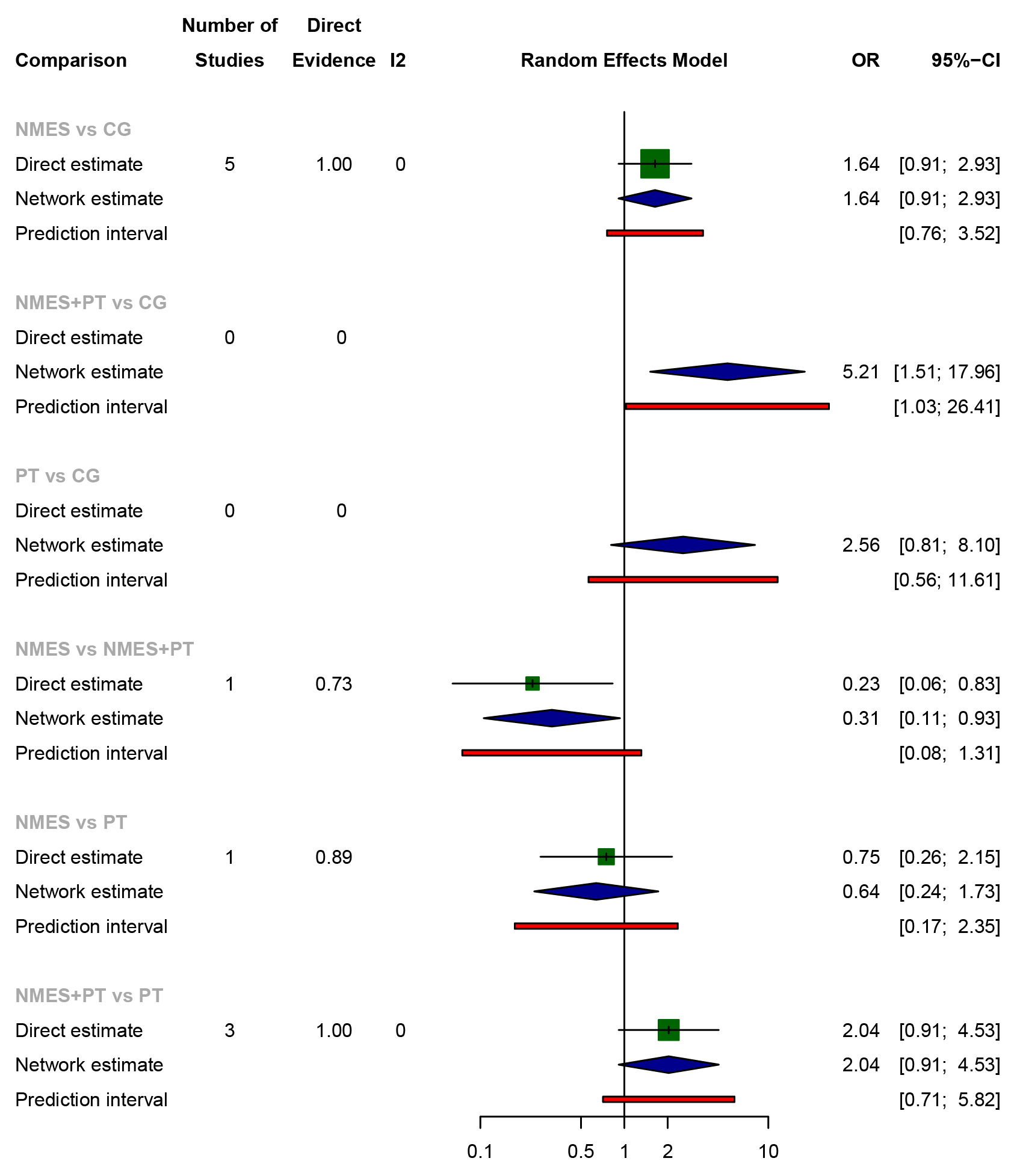


**Supplementary Fig. 5c** Forest plot of pairwise meta-analysis, network meta-analysis and predictive interval for extubation success rate from sensitivity analyses. The pairwise meta-analysis indicated that the combination of NMES with PT displayed a slightly higher success rate than NMES alone. The network meta-analysis reinforced the superiority of the NMES+PT over NMES, and the same for NMES over CG for extubation successs rate. The predictive interval plots revealed significant heterogeneity in the network meta-analysis when comparing the extubation success rate between NMES+PT and NMES alone.

**
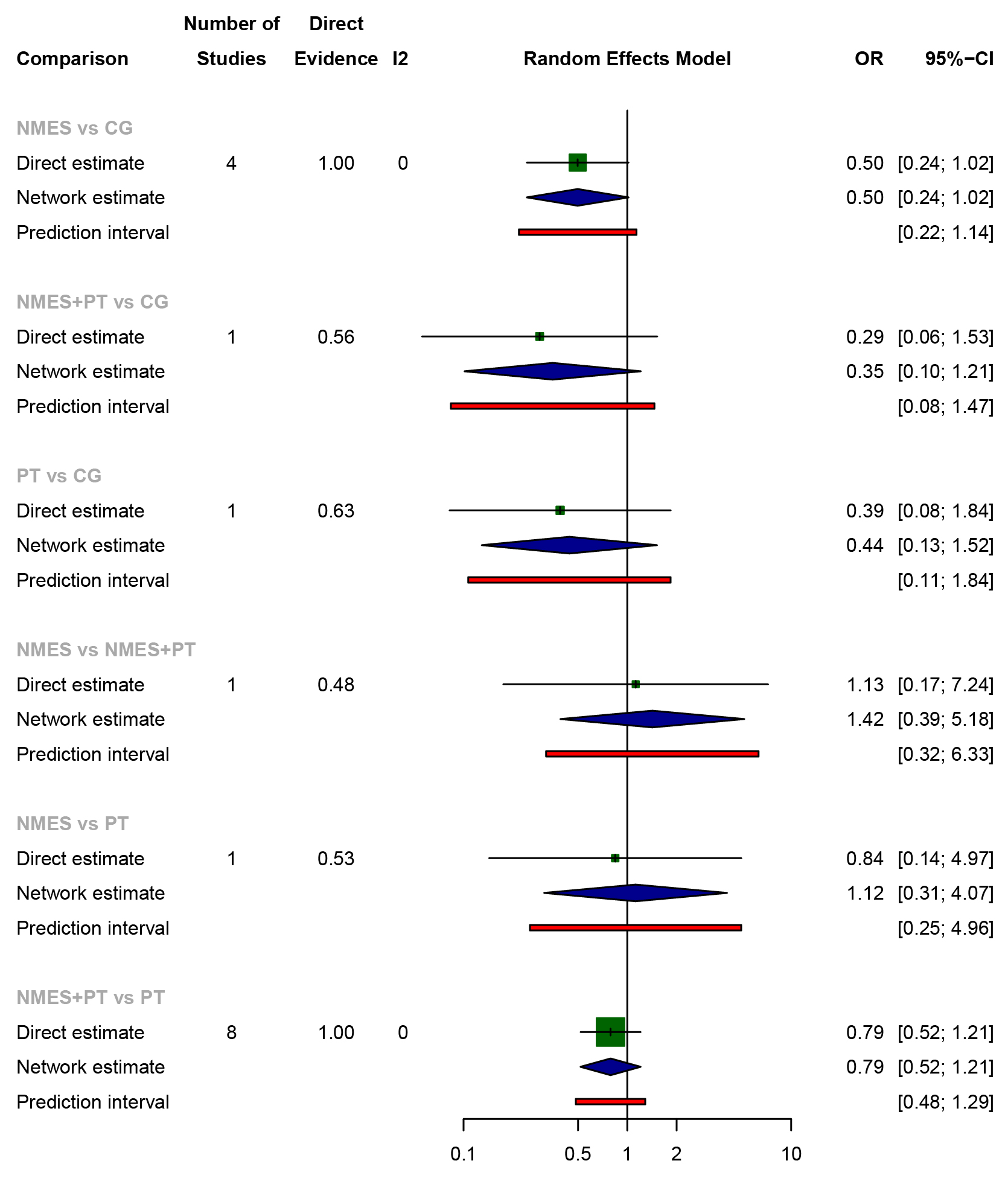
**

**Supplementary Fig. 5d** Forest plot of pairwise meta-analysis, network meta-analysis and predictive interval for mortality from sensitivity analyses. Both the pairwise meta-analysis and the network meta-analysis revealed no significant differences among the four treatments for mortality. Predictive interval plots suggested no significant heterogeneity in the network meta-analysis among the comparisons for mortality.
